# Nanoconfinement‐Steered Molecular Preorganization Enables Efficient Monoethanolamine Degradation through Electronically Modulated High‐Valent Iron–Oxo Pathways

**DOI:** 10.1002/advs.202514979

**Published:** 2025-11-25

**Authors:** Lin Zhang, Qin Dai, Xinyue Chen, Hang Yuan, Lei Xing, Zhimo Fang, Xiuze Li, Xiangke Wang, Lihui Zhang, Junping Zhao, Qiangwei Li, Lidong Wang

**Affiliations:** ^1^ MOE Key Laboratory of Resources and Environmental Systems Optimization College of Environmental Science and Engineering North China Electric Power University Beijing 102206 P. R. China; ^2^ National Engineering Research Center of New Energy Power Generation North China Electric Power University Beijing 102206 P. R. China; ^3^ Institute of Energy Resources Hebei Academy of Sciences Shijiazhuang 050081 P. R. China

**Keywords:** active species transition, advanced oxidation processes, confinement strategy, high‐valent iron–oxo, wastewater remediation

## Abstract

While nanoconfinement effectively stabilizes ultrasmall metal nanoparticles to enhance peroxymonosulfate (PMS) activation in Fenton‐like systems, precise control over reaction pathways remains challenging. To address this, ultrasmall UiO‐66─ZrFe nanoparticles within a π‐electron‐enriched asymmetric graphene aerogel (GA) are immobilized. This designed confinement architecture not only redirects PMS‐activated monoethanolamine (MEA) degradation from nonselective radical oxidation to a dominant high‐valent iron–oxo (Fe(IV)═O) pathway but, more importantly, enables a synergistic mechanism coupling molecular preorganization with proton–electron coregulation. Specifically, spatial constraints within UiO‐66─ZrFe cages enable MEA to orient its amino group (─NH_2_) toward Fe(IV)═O species. This configuration promotes electrophilic attack by Fe(IV)═O on the lone‐pair electrons in ─NH_2_, simultaneously triggering intramolecular C─N bond cleavage with concomitant proton release. Liberated protons are electrostatically stabilized on the π‐conjugated GA network, establishing synergistic coupling with Fe─PMS coordination. This interfacial synergy facilitates directional electron transfer from Fe sites to the carbon matrix and results in regioselective catalysis, enhancing MEA degradation kinetics by 4.85‐fold. Practical viability for wastewater remediation is confirmed through life cycle assessment and extended operation. This strategy establishes a generalized design principle for precise reaction control via the orchestration of coupled spatial and electronic effects within engineered microenvironments.

## Graphical abstract

Precise modulation of reaction pathways remains an unresolved challenge in peroxymonosulfate‐activated advanced oxidation processes. The spatial preorganization strategy in engineered confinement architecture enables regioselective bond cleavage in monoethanolamine via synergistic proton–electron transfer, redirecting its degradation from nonselective radicals toward a dominant high‐valent iron–oxo pathway and thereby establishing programmable microenvironments for precision catalysis.

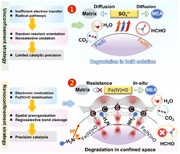

## 1 Introduction

Water pollution presents an escalating global crisis, profoundly threatening human health and undermining the integrity of aquatic ecosystems worldwide.^[^ ^1^, ^2^ ^]^ Nitrogen‐containing organic compounds, which constitute over 65% of identified total organic compounds across environmental matrices, have emerged as critical remediation priorities due to their recalcitrant chemical nature and multifaceted ecological hazards.^[^ ^3^ ^]^ While conventional peroxymonosulfate (PMS)‐activated Fenton‐like advanced oxidation processes (AOPs) harness reactive oxygen species (ROS) for pollutant decomposition,^[^ ^4^, ^5^ ^]^ their effectiveness remains fundamentally constrained by dual operational limitations: the severe mass transfer inefficiency stemming from radicals’ vanishingly short lifetimes (10^−6^–10^−9^ s) and nanometer‐scale diffusion distances (<200 nm), coupled with inherent nonselective oxidation mechanisms that render them vulnerable to rapid quenching by ubiquitous background constituents.^[^ ^6^ ^]^ These intrinsic deficiencies precipitate substantial efficiency deterioration in real‐world aqueous systems, driving urgent demand for innovative catalytic solutions.^[^ ^7^ ^]^

Nanoconfinement engineering marks a paradigm shift to transcend these barriers,^[^ ^8^, ^9^ ^]^ creating precise spatial constraints that concentrate reactants while simultaneously reconfiguring electronic configurations to modulate reaction coordinates.^[^ ^10^, ^11^ ^]^ Representative demonstrations include the confinement of 2 nm Fe_2_O_3_ within 7 nm carbon nanotube cavities, elevating methylene blue degradation kinetics by 22.5‐fold through enhanced mass transfer,^[^ ^12^ ^]^ alongside FeOCl encapsulated in 20 nm membrane pores that amplify *p*‐chlorobenzoic acid decomposition rates by three orders of magnitude via intensified molecular collisions.^[^ ^13^ ^]^ Most notably, beyond augmenting mass transfer, this approach enables ingenious reaction selectivity control, exemplified by Fe(III) systems confined within graphene aerogel (GA) that redirect phenol degradation from chaotic ring cleavage toward controlled oligomerization pathways.^[^ ^14^ ^]^ Particularly significant is the exceptional catalytic profile of high‐valent metal species such as high‐valent iron–oxo (Fe(IV)═O), whose persistence and molecular recognition capacity toward electron‐donating functional groups establish superior selectivity for alkanolamine oxidation (e.g., monoethanolamine, MEA)—starkly contrasting with the indiscriminate reactivity patterns of radical species.^[^ ^15^, ^16^, ^17^, ^18^ ^]^ However, existing research has yet to establish a theoretical framework for critical scientific issues such as the dynamic regulation mechanisms of electron transfer pathways in confined catalysts—a deficiency that exacerbates the instability of active centers and compromises oxidative precision, severely hampering practical application.^[^ ^19^, ^20^, ^21^ ^]^

Herein, we propose a proton–electron coregulation strategy, based on GA‐confined ultrasmall UiO‐66─ZrFe catalyst, which aims to overcome the aforementioned bottlenecks by driving spatial preorganization to achieve regioselective cleavage of MEA.^[^ ^3^ ^]^ The core mechanism resides in the integration of UiO‐66─ZrFe molecular cages with the π‐conjugated GA substrate, enabling the spatially constrained cavities to leverage the synergistic effect of geometric confinement and electrostatic guidance. This synergistic effect forces the preorganization of MEA molecules within the highly ordered rigid cavity structure. Consequently, the amino group (─NH_2_) is precisely positioned proximal to the electrophilic Fe(IV)═O site located at the cage vertex, thereby triggering the regioselective intramolecular C─N bond cleavage of MEA. Furthermore, the electron‐rich Fe(IV)═O center mediates a self‐sustaining catalytic cycle: the lone pair on the nitrogen atom synchronously drives both the reduction of Fe(IV)═O and the intramolecular dehydrogenation of the ─NH_2_ moiety, liberating protons. These liberated protons are instantaneously chemically adsorbed onto the adjacent GA, inducing localized electron deficiency which disrupts the symmetry of the π‐conjugated system. This symmetry breaking synergistically couples with Fe (3d)─PMS (2p) orbital hybridization, cooperatively mediating the directional electron transfer from the PMS antibonding orbital to the carbon matrix. Ultimately, the spatial confinement effect suppresses the disordered diffusion of reactants. This suppression, combined with the proton–electron synergistic interaction, achieves the sustained regeneration of the Fe(IV)═O species. Simultaneously, this integrated mechanism effectively suppresses side reactions, significantly enhancing both the control precision and the efficiency of the catalytic pathway. This work aims to establish a new design paradigm that moves beyond simple confinement effects by demonstrating the critical role of coupling reactant preorganization with interfacial proton–electron dynamics for achieving precise reaction control.

## 2 Results and Discussion

### 2.1 Characterizations

**Figure 1:**
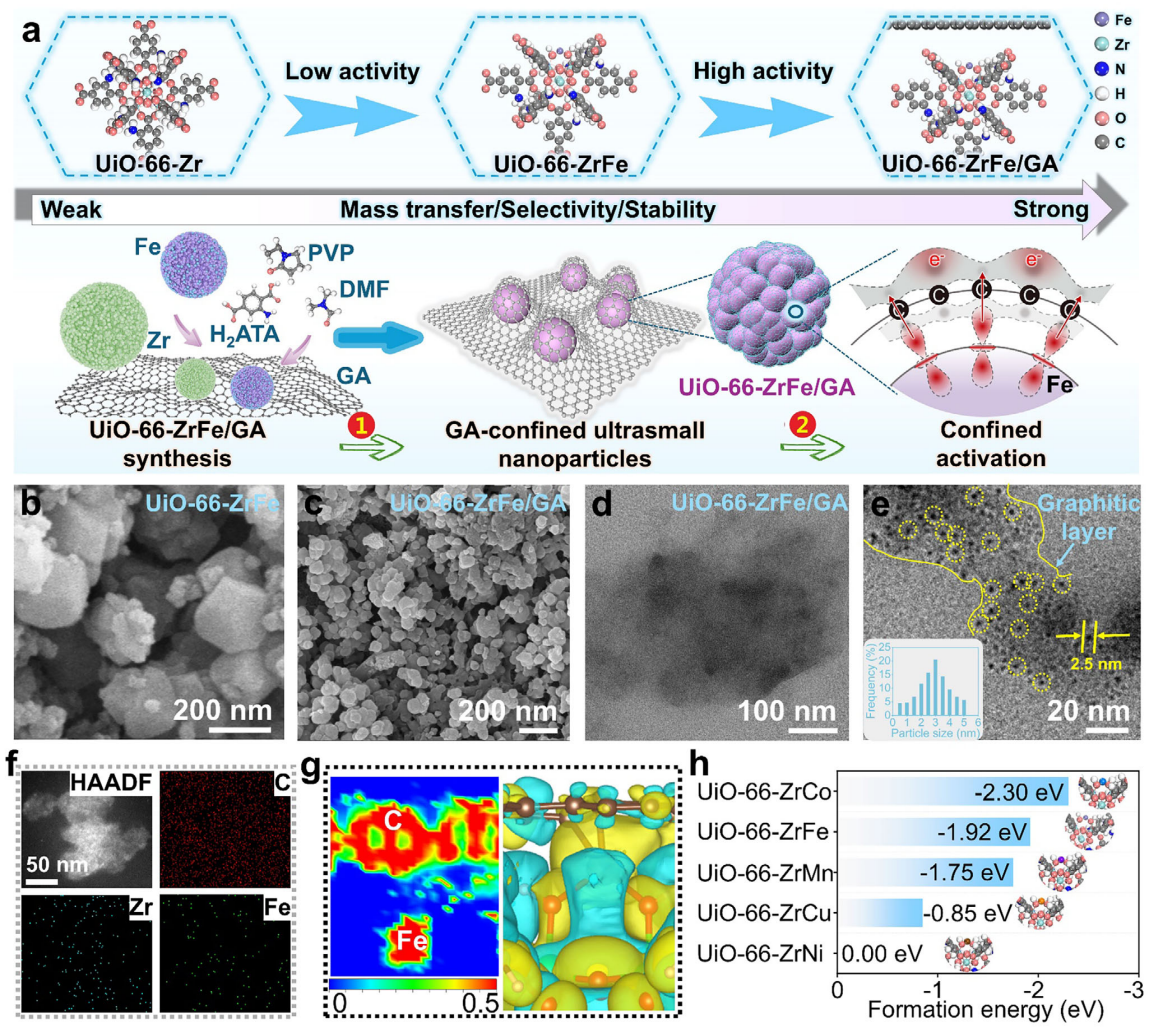
a) Molecular structure of the catalysts and schematic illustration of the confinement strategy in UiO‐66─ZrFe/GA; b) SEM image of UiO‐66─ZrFe; c–e) TEM and HRTEM images of UiO‐66─ZrFe/GA; f) HAADF‐STEM elemental mapping of UiO‐66─ZrFe/GA; g) electron localization function (ELF) and charge density difference (CDD) analyses for UiO‐66─ZrFe/GA; h) calculated formation energies of the catalysts.

Synthetic procedures yielded multiple metal–organic frameworks (MOFs), namely UiO‐66─Zr, UiO‐66─ZrFe, and UiO‐66─ZrFe/GA (**Figure** **1**a; Figure S1, Supporting Information). Morphological characterization via scanning electron microscopy (SEM), transmission electron microscopy (TEM), and high‐resolution TEM (HRTEM) revealed distinct structural features of GA, UiO‐66─ZrFe, and UiO‐66─ZrFe/GA. Pristine GA exhibited a defined morphology (Figure S2a, Supporting Information), while UiO‐66─ZrFe formed regular nanoparticles with dimensions of tens of nanometers (Figure 1b; Figure S2b, Supporting Information). For UiO‐66─ZrFe/GA composites (Figure 1c–e), the GA substrate was clearly identifiable, with uniformly dispersed UiO‐66─ZrFe particles (≈2.5 nm diameter). This significant reduction in MOF particle size is attributed to GA‐mediated nucleation control and spatial confinement during growth, demonstrating effective nanoparticle immobilization within the amorphous GA architecture.^[^ ^22^, ^23^, ^24^ ^]^ Elemental mapping via high‐angle annular dark‐field scanning TEM (HAADF‐STEM) confirmed the homogeneous coexistence of Fe and Zr (Figure 1f). Textural properties of UiO‐66─ZrFe/GA—including Barrett–Joyner–Halenda pore volume, Brunauer–Emmett–Teller surface area (175.18 m^2^ g^−1^), and average pore diameter (6.54 nm)—are summarized in Table S2 (Supporting Information). Nitrogen adsorption–desorption isotherms displayed a Type IV profile with H_3_ hysteresis (Figure S3, Supporting Information), indicating a mesoporous structure with irregular pore size distribution. The substantial specific surface area facilitates pollutant adsorption capacity, and the developed pore structure enhances small‐molecule accessibility.

In Figure 1g, the concomitant reduction in electron localization function (ELF) at the Fe–GA interfaces signifies the presence of a nonbonding confinement cavity. This cavity creates spatially defined zones for reactant preconcentration. Charge density difference (CDD) provides clear evidence of electron transfer from Fe sites to GA. The synergistic interplay of this distinct electronic environment and geometric architecture promotes efficient charge redistribution and localized reactant enrichment, thereby enhancing catalytic activation. The calculated formation energies of catalysts incorporating diverse metal species exhibit distinct thermodynamic stability trends. As evidenced in Figure 1h, the formation energy of UiO‐66─ZrFe ranks second only to that of Co among the investigated metals, demonstrating its exceptional thermodynamic stability.

**Figure 2:**
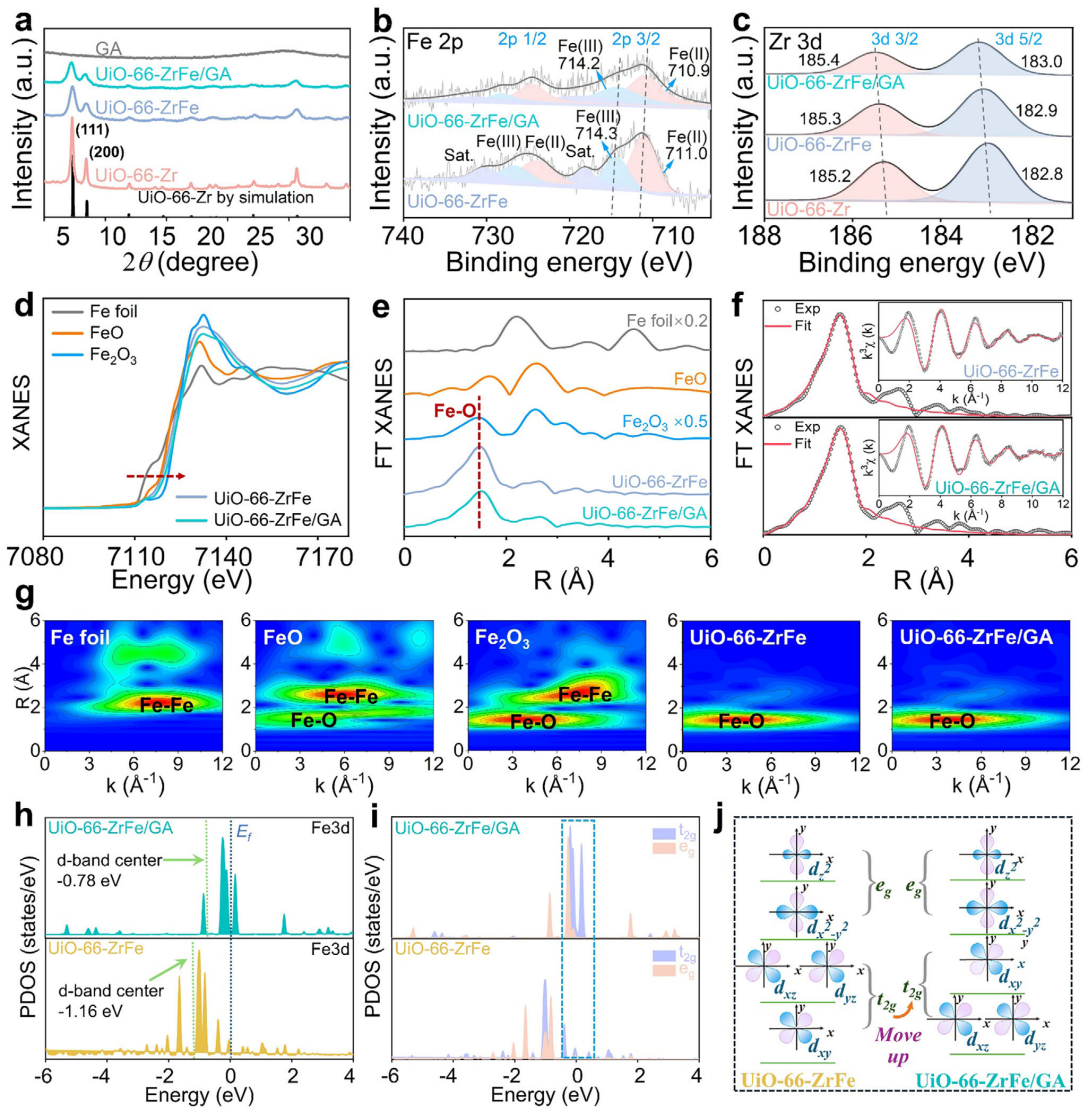
a) XRD patterns; XPS spectra of b) Fe 2p and c) Zr 3d; d) XANES and e) FT‐EXAFS spectra at the Fe K‐edge; f) EXAFS fitting curves for UiO‐66─ZrFe and UiO‐66─ZrFe/GA; g) wavelet transform analysis of the EXAFS; h,i) partial density of states (PDOS) for UiO‐66─ZrFe and UiO‐66─ZrFe/GA; j) schematic representation of the splitting of d‐orbitals into e_g_ and t_2g_ manifolds.

The X‐ray diffraction (XRD) patterns of the four samples (**Figure** **2**a) demonstrate that UiO‐66─Zr exhibits characteristic peaks consistent with literature.^[^ ^25^, ^26^ ^]^ Incorporation of Fe in UiO‐66─ZrFe preserves the MOF crystalline structure, while UiO‐66─ZrFe/GA maintains this framework as evidenced by distinct (200) and (111) diffraction peaks, albeit with attenuated intensities.^[^ ^27^ ^]^ X‐ray photoelectron spectroscopy (XPS) analysis (Figure 2b,c) elucidates the chemical states of Fe and Zr. Deconvolution of the Fe 2p spectra (Figure 2b) reveals four peaks in UiO‐66─ZrFe, with binding energies at 711.0 and 714.3 eV explicitly assigned to Fe(II) 2p 3/2 and Fe(III) 2p 3/2 spin‐orbitals, respectively.^[^ ^28^ ^]^ The direct comparison between UiO‐66─ZrFe and UiO‐66─ZrFe/GA unequivocally isolates the specific contribution of the graphene aerogel matrix. The observed negative binding energy shift in UiO‐66─ZrFe/GA is a direct consequence of the GA confinement, which enhances the local electron density around the Fe centers. This electronic modulation facilitated by GA is pivotal for the subsequent activation of PMS.^[^ ^29^ ^]^ Conversely, Zr 3d spectra (Figure 2c) show marginal positive shifts in the Zr(IV) 3d 5/2 and 3d 3/2 orbitals for UiO‐66─ZrFe/GA, suggesting reduced electron density.^[^ ^30^ ^]^ The direction and magnitude of these binding energy shifts for Zr were robust and reproducible across multiple independent sample batches (Figure S4, Supporting Information), unequivocally confirming that they are genuine electronic effects and not artifacts. The observed concomitant increase in electron density at the Fe sites and decrease at the Zr nodes is consistent with an interfacial charge redistribution within the UiO‐66 framework, likely facilitated by the highly conductive graphene aerogel matrix. This electronic “spillover” effect preferentially enriches the active Fe centers with electron density, thereby fine‐tuning their catalytic properties.

X‐ray absorption fine structure (XAFS) analysis was employed to probe the atomic coordination environment of Fe. The X‐ray absorption near‐edge structure (XANES) spectra in Figure 2d now include a direct comparison between UiO‐66─ZrFe and UiO‐66─ZrFe/GA. Both samples exhibit a right‐shifted absorption edge relative to the FeO reference, confirming an elevated Fe valence state.^[^ ^15^ ^]^ Consistent with this finding, the Fourier‐transform extended X‐ray absorption fine structure (EXAFS) (Figure 2e) for both materials exhibits a prominent Fe─O coordination peak at ≈1.5 Å, signifying Fe anchoring at Zr_6_‐oxo cluster peripheries rather than node doping.^[^ ^27^ ^]^ Beyond the first‐shell Fe─O coordination, a weaker feature is observed in the *R*‐space range of ≈2–3 Å (Figure 2e). Comprehensive fitting confirms that this feature does not originate from Fe─Fe or Fe─Zr scattering, as such metal–metal paths are structurally precluded in the atomically dispersed UiO‐66 matrix where Fe sites are isolated within the [Zr_6_O_4_(OH)_4_] clusters. Instead, this contribution is best assigned to single‐scattering paths from the carbon atoms in the terephthalate linkers, which constitute a secondary coordination environment around the Fe centers.

Structural refinement of the EXAFS fitting curves (Figure 2f; Table S3, Supporting Information) yields a similar first‐shell coordination number for Fe in both catalysts. It is noteworthy that this finding forms a profoundly complementary evidence with the observed XPS results, where a negative binding energy shift for Fe is detected in UiO‐66─ZrFe/GA. The high sensitivity of XPS to the local electron density at the surface reveals electron enrichment at the Fe active sites induced by the GA confinement effect. By contrast, XANES, which probes the bulk‐average valence state, confirms the preservation of the fundamental Fe coordination structure. To further resolve the coordination environment and rule out the presence of Fe clusters, wavelet transform EXAFS analysis was conducted for both UiO‐66─ZrFe and UiO‐66─ZrFe/GA (Figure 2g). The wavelet transform plots for both materials display a single, well‐defined intensity maximum at a position in *k*‐space corresponding solely to Fe─O coordination. No intensity contours are observed in the higher *k*‐range where Fe─Fe scattering would be expected. The identical features between UiO‐66─ZrFe and UiO‐66─ZrFe/GA unambiguously confirm that the atomic‐level dispersion of Fe sites and their local Fe─O coordination geometry are intrinsic properties of the Fe‐doped UiO‐66 structure and are preserved upon integration with the GA matrix. This result reinforces that the GA's role is to provide a superior environment for catalysis without altering the fundamental atomic‐scale coordination of the active metal centers.

The partial density of states (PDOS) for UiO‐66─ZrFe and UiO‐66─ZrFe/GA are presented in Figure 2h. Compared to UiO‐66─ZrFe, UiO‐66─ZrFe/GA exhibits enhanced electronic occupied states near the Fermi level (*E*_f_), implying greater availability of electrons for catalytic PMS activation facilitated by GA. Concurrently, the d‐band center shifts from −1.16 eV in UiO‐66─ZrFe to −0.78 eV in UiO‐66─ZrFe/GA, closer to the *E*_f_. This shift suggests stronger PMS chemisorption on UiO‐66─ZrFe/GA. Notably, GA incorporation induces an upward shift of the entire Fe t_2g_ orbital in UiO‐66─ZrFe/GA (Figure 2i,j), which promotes catalytic reactivity. Furthermore, GA incorporation induces degeneracy lifting of the d*_xz_* and d*_yz_* orbitals at low energy levels, implying minimal structural distortion of the catalyst framework. Collectively, GA optimizes the Fe 3d orbital configuration, enhances electron redistribution, and accelerates PMS activation, thereby enabling efficient MEA degradation.

### 2.2 Dual Enhancements of Mass Transfer and Selectivity

**Figure 3:**
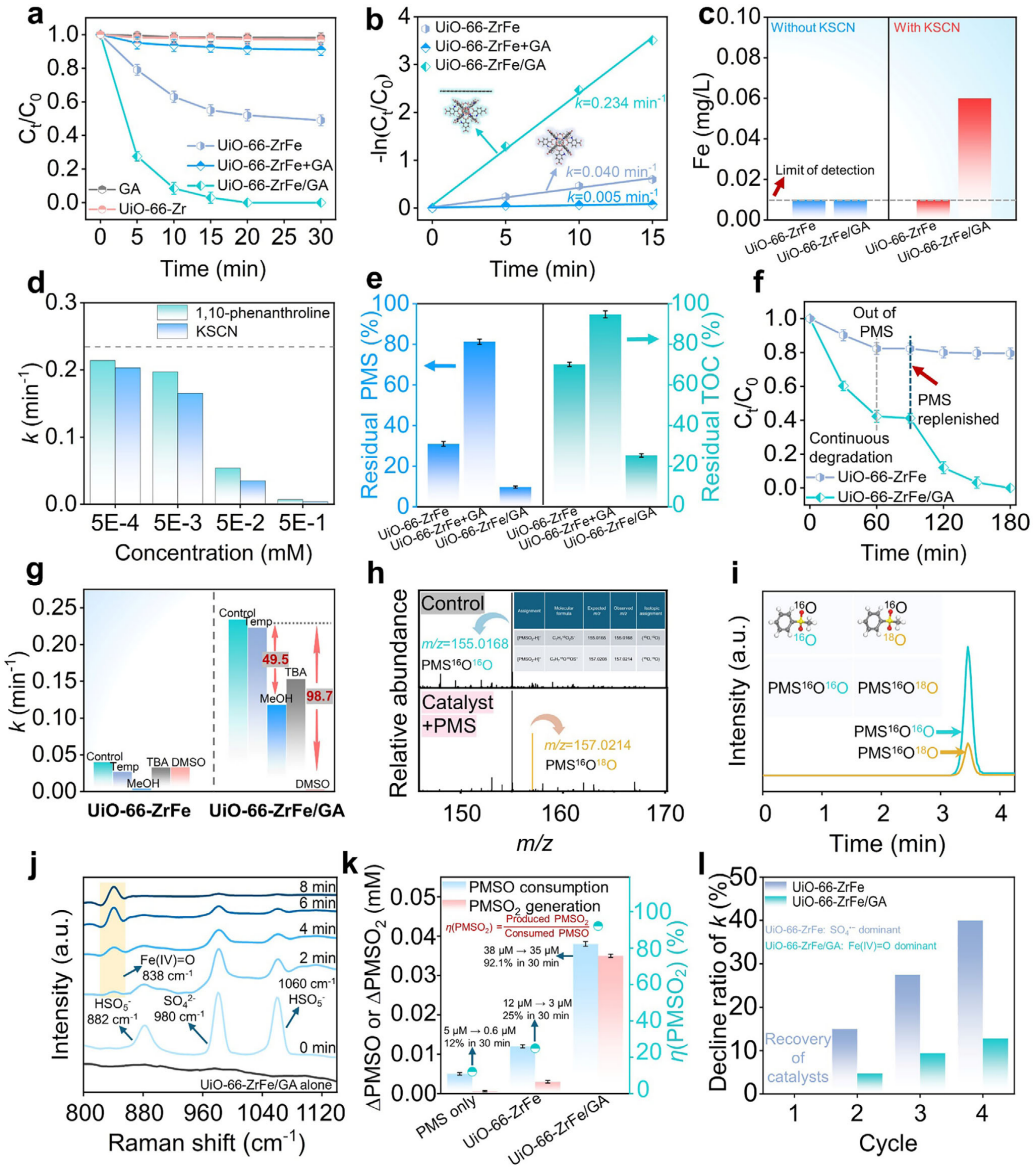
a) MEA degradation efficiency via PMS activation across different catalytic systems. b) The corresponding pseudo‐first‐order kinetic fitting. c) Comparative Fe leaching analysis for UiO‐66─ZrFe and UiO‐66─ZrFe/GA in the presence and absence of KSCN following PMS addition. d) Scavenging tests targeting Fe(II) and Fe(III) species. e) Residual PMS (%) and residual total organic carbon (TOC) (%), residual PMS (%) was calculated as (*C*/*C*_0_) × 100%, where *C* and *C*_0_ are the residual and initial concentrations of PMS determined by iodometric titration; residual TOC (%) was calculated as (TOC/TOC_0_) × 100%, where TOC and TOC_0_ are the residual and initial TOC concentrations. f) Long‐term MEA degradation performance. g) Effects of radical and nonradical scavengers on degradation efficiency. h) High‐resolution mass spectrometry detection of PMS^16^O^18^O generated in H_2_^18^O. i) Extracted ion chromatograms of PMS^16^O^16^O and PMS^16^O^18^O species formed from PMS^16^O^16^O within the UiO‐66─ZrFe/GA + PMS system in H_2_^18^O. j) Time‐series in situ Raman spectra collected during the reaction of UiO‐66─ZrFe/GA with PMS. k) The stoichiometric evaluation of the Fe(IV)═O pathway probed by methyl phenyl sulfoxide (PMSO) oxidation. The yield of methyl phenyl sulfone (PMSO_2_), denoted as *η*(PMSO_2_), is defined by (produced PMSO_2_/consumed PMSO) × 100%. The actual concentration of PMSO_2_ reached 35 µm from an initial PMSO concentration of 38 µm, corresponding to a yield of 92.1% within. The global reaction conditions were (PMS) = 2 mm and catalyst loading = 0.2 g L^−1^. l) Evaluation of the catalytic reusability of UiO‐66─ZrFe/GA over four consecutive cycles. The gradual decrease in the *k* is attributed to operational factors such as catalyst fouling or minor structural alterations under rigorous reaction conditions. It is important to emphasize that the Fe(IV)═O species is a catalytic intermediate regenerated in each cycle. The excellent retention of the catalyst's crystalline structure is confirmed by postreaction XRD and inductively coupled plasma (ICP) analysis, collectively affirming the robust stability of the catalyst. Data points represent the mean values from three independent experimental replicates (*n* = 3), and error bars indicate the standard deviation.

The catalytic activities of the synthesized catalysts were evaluated through PMS activation for MEA degradation. In catalyst‐free control experiments, PMS alone exhibited limited efficacy for MEA removal, achieving only 4.4% elimination within 30 min (Figure S5, Supporting Information). Furthermore, the five investigated catalysts demonstrated negligible MEA adsorption capacity over the same duration in the absence of PMS (Figure S5, Supporting Information). Comparative analysis of degradation performance (**Figure** **3**a) revealed the superior activity of the UiO‐66─ZrFe + PMS and UiO‐66─ZrFe/GA + PMS systems against controls (GA alone, UiO‐66─Zr alone, and a physical mixture of UiO‐66─ZrFe + GA). Upon PMS introduction, GA and UiO‐66─Zr individually induced minimal MEA degradation (1.8% and 3.0%, respectively), indicating their inherent inertness. By contrast, UiO‐66─ZrFe catalyzed significant degradation, removing 51.0% of MEA within 30 min. However, the physical combination of UiO‐66─ZrFe with GA failed to enhance catalytic performance, yielding only 9.1% degradation. Remarkably, the composite catalyst UiO‐66─ZrFe/GA (0.2 g L^−1^) facilitated complete MEA degradation within 30 min. Kinetic analysis, fitting the degradation data to a pseudo‐first‐order model (Figure 3b), determined a rate constant (*k*) of 0.234 min^−1^ for the UiO‐66─ZrFe/GA + PMS system, representing a 4.85‐fold enhancement over the UiO‐66─ZrFe + PMS system (*k* = 0.040 min^−1^). As illustrated in Figure S6 (Supporting Information), degradation efficiency and kinetics were strongly dependent on catalyst loading, PMS dosage, and initial MEA concentration (detailed discussion in Text S10 in the Supporting Information). Furthermore, the robustness of the UiO‐66─ZrFe/GA + PMS system was demonstrated across a range of initial pH conditions, exhibiting marked resistance to interference (Figure S7, Supporting Information).

To validate the presence and elucidate the role of confined microenvironments, Fe leaching was assessed in the UiO‐66─ZrFe + PMS and UiO‐66─ZrFe/GA + PMS systems (Figure 3c). Negligible Fe leaching was observed in the filtrate of both systems without KSCN addition, with concentrations below the method's limit of detection (0.01 mg L^−1^). The introduction of KSCN, which forms a specific colored complex with Fe^3+^ ions, was essential for detecting Fe that remains spatially confined within the catalyst's microstructure near the solid–liquid interface rather than being freely released into the bulk solution. This assay revealed an increase in detectable Fe specifically for the UiO‐66─ZrFe/GA composite, providing direct experimental evidence for the confined microenvironment.^[^ ^31^ ^]^ The GA matrix thus facilitated reactive species generation while effectively suppressing Fe leaching, as the framework prevented the migration of Fe species beyond the solid–liquid interface. The KSCN complexation reaction successfully accessed and detected this non‐leached, confined Fe. By contrast, the UiO‐66─ZrFe + PMS system showed no detectable Fe leaching even in the presence of KSCN, consistent with its inferior catalytic performance.^[^ ^31^ ^]^

Further mechanistic insight into the origin of active species was obtained through ligand addition control experiments. Specifically, equimolar concentrations of 1,10‐phenanthroline (Fe(II) scavenger) and KSCN (Fe(III) scavenger) were employed.^[^ ^31^ ^]^ As shown in Figure 3d, inhibition intensified with increasing scavenger concentration, confirming the critical involvement of both Fe(II) and Fe(III) active sites. To rule out the potential nonspecific site‐blocking effect of 1,10‐phenanthroline, a parallel experiment was conducted using ferrozine, an alternative Fe(II)‐specific chelator with a distinct molecular structure (Figure S8, Supporting Information). The nearly identical inhibitory effect observed with ferrozine confirms that the activity loss is indeed due to the specific complexation of Fe(II) rather than physical site blockage. The results collectively paint a coherent picture where the catalytic process necessitates a continuous Fe(II)/Fe(III) redox cycle. The sequestration of either valence state effectively halts this cycle, leading to a complete cessation of PMS activation and consequently the observed near‐total inhibition of MEA degradation. The confinement environment in UiO‐66─ZrFe/GA serves to optimize this intrinsic Fe cycle while simultaneously suppressing Fe leaching. The UiO‐66─ZrFe/GA + PMS system demonstrated efficient PMS activation (90.3% utilization rate, Figure 3e) and significantly enhanced total organic carbon (TOC) removal (74.7%), markedly surpassing the unconfined UiO‐66─ZrFe + PMS system (29.9% TOC removal). This highlights the system's proficiency in achieving efficient organic pollutant mineralization.^[^ ^32^, ^33^ ^]^ Continuous MEA degradation over 180 min was sustained using UiO‐66─ZrFe/GA (Figure 3f), with PMS replenishment overcoming initial stagnation. Conversely, UiO‐66─ZrFe exhibited limited activity, minimally improved by PMS addition. The stability and structural integrity of the UiO‐66─ZrFe/GA catalyst were rigorously confirmed through comprehensive postreaction characterization. Postreaction XRD confirmed structural integrity (Figure S9, Supporting Information), while minimal Fe leaching (8.5 µg L^−1^ after 30 min, Figure S10, Supporting Information) and comparable elemental composition between fresh and used catalysts (inductively coupled plasma (ICP) analysis, Table S4, Supporting Information) demonstrate robustness. Homogeneous Fe^2+^/Fe^3+^ ions (0.01 mg L^−1^) exhibited negligible degradation activity (Figure S11, Supporting Information), confirming heterogeneous catalysis dominates the UiO‐66─ZrFe/GA + PMS system. These findings collectively demonstrate the significant potential of the UiO‐66─ZrFe/GA + PMS system for treating MEA‐contaminated wastewater.

### 2.3 Catalytic Pathway Transition Triggered by Confinement Strategy

Quenching experiments were conducted to elucidate the contributions of distinct ROS to the degradation efficacy in both the UiO‐66─ZrFe + PMS and UiO‐66─ZrFe/GA + PMS systems. As demonstrated in Figure 3g and Figure S12 (Supporting Information), introduction of methanol (MeOH), a scavenger for both ^•^OH and SO_4_^•−^,^[^ ^34^, ^35^, ^36^ ^]^ reduced MEA degradation to 6.8% in the UiO‐66─ZrFe + PMS system, indicating SO_4_^•−^ predominance. Conversely, addition of *tert*‐butanol, a selective ^•^OH scavenger,^[^ ^37^, ^38^ ^]^ yielded 40.2% degradation, further confirming the secondary role of ^•^OH. In the UiO‐66─ZrFe/GA + PMS system, MeOH addition resulted in a 49.5% reduction in the *k*. Significantly, when dimethyl sulfoxide was employed to quench Fe(IV)═O,^[^ ^39^, ^40^ ^]^ degradation plummeted to 5%, accompanied by a 98.7% reduction in the *k* (Figure 3g; Figure S13, Supporting Information). These results verify the dominant contributions of Fe(IV)═O to MEA degradation within the confined catalytic microenvironment. Electron paramagnetic resonance (EPR) spectroscopy was employed to identify radical species generated in the UiO‐66─ZrFe + PMS and UiO‐66─ZrFe/GA + PMS systems (Figure S14, Supporting Information). Using 5,5‐dimethyl‐1‐pyrroline N‐oxide as a spin trap, a pronounced EPR signal characteristic of SO_4_^•−^ and ^•^OH was observed for UiO‐66─ZrFe + PMS. This signal was significantly attenuated in the UiO‐66─ZrFe/GA + PMS system, suggesting that SO_4_^•−^ generation predominantly occurs in an unconfined reaction environment.

The identification of the reaction product as methyl phenyl sulfone (PMSO_2_) and the evidence for oxygen atom transfer from the Fe(IV)═O species were unequivocally confirmed by high‐resolution mass spectrometry. As illustrated in Figure 3h, the deprotonated ion [M─H]^−^ of the unlabeled PMSO_2_ was detected at *m/z* = 155.0168, which matches excellently with the theoretical value for C_7_H_7_^16^O_2_S^−^ (theoretical *m/z* = 155.0165, inset table).^[^ ^41^ ^]^ Crucially, no PMS^16^O^18^O was detected in a control experiment containing only PMS^16^O^16^O in H^18^O, confirming the absence of direct oxygen exchange.^[^ ^42^ ^]^ When the reaction was conducted in an H_2_^18^O matrix, the corresponding ^18^O‐labeled product was observed at *m/z* = 157.0214, assigned to C_7_H_7_^16^O^18^OS^−^ (theoretical *m/z* = 157.0208). The coexistence of both species is vividly demonstrated by the two distinct peaks in the extracted ion chromatograms (Figure 3i). The clear presence of the ^18^O‐labeled PMSO_2_ provides direct and compelling evidence that the oxygen atom in the Fe(IV)═O exchanges with solvent water via an oxygen atom transfer pathway, thereby constituting the primary Fe(IV)═O mechanism for PMS activation in the UiO‐66─ZrFe/GA + PMS system. The time‐series in situ Raman spectroscopy was employed in Figure 3j to dynamically probe the reaction mechanism. The evolution of the integrated peak areas of Figure 3j provides direct insight, as shown in Figure S15a (Supporting Information). A monotonic decrease in the PMS bands confirms the continuous consumption of the oxidant. Concurrently, the band at 838 cm^−1^ exhibited a rapid increase in intensity reaching a steady‐state plateau that was maintained throughout the reaction. This distinct profile reveals that the Fe(IV)═O intermediate attains a dynamic steady‐state concentration on the catalyst surface balancing its formation via PMS heterolysis against its inherent decay. The assignment of this band to Fe(IV)═O was unequivocally confirmed by an H_2_^18^O isotope‐labeling experiment in Figure S15b (Supporting Information). The characteristic Raman peak shifted from 838 cm^−1^ in H_2_^16^O to 827 cm^−1^ in H_2_^18^O. This redshift is a direct signature of the isotopic mass effect on the Fe═O bond vibration providing definitive proof of the species identity.^[^ ^43^ ^]^ The ability of the UiO‐66─ZrFe/GA architecture to stabilize a detectable population of this potent oxidant underscores a key advantage of our confinement strategy.

Figure S16 (Supporting Information) quantifies the oxidation of methyl phenyl sulfoxide (PMSO) and the resultant PMSO_2_ generation in the UiO‐66─ZrFe/GA + PMS system, revealing that 38 µmol L^−1^ of PMSO was consumed to yield 35 µmol L^−1^ of PMSO_2_ within 30 min, maintaining a molar conversion ratio (*η*(PMSO_2_)) of over 91%. For comparison, PMS alone cannot oxidize PMSO to produce PMSO_2_, and UiO‐66─ZrFe + PMS converted limited PMSO with a yield of only 25% (Figure 3k). To quantitatively assess the contribution of the Fe(IV)═O pathway relative to the radical pathways, a competition experiment was performed. The specific Fe(IV)═O probe, PMSO, and the pollutant, MEA, were introduced concurrently into the UiO‐66─ZrFe/GA + PMS system. A decrease in the generation of PMSO_2_ was observed compared to the system without MEA (Figure S17, Supporting Information). This inhibition occurs because both PMSO and MEA compete for the available Fe(IV)═O species. Since PMSO oxidation is highly specific to Fe(IV)═O, the suppression of its conversion provides direct evidence that a substantial fraction of the oxidizing capacity is channeled through the Fe(IV)═O intermediate for MEA degradation. This result quantitatively confirms that Fe(IV)═O acts as the dominant oxidant. Subsequently, the catalytic stability of UiO‐66─ZrFe/GA was investigated. It demonstrated exceptional reusability, showing merely a minor decrement in its degradation rate after undergoing four cycles while still achieving complete removal of MEA within 30 min (as illustrated in Figure S18 in the Supporting Information). Conversely, UiO‐66─ZrFe exhibited a comparably poorer recycling performance, with a notable 40% reduction in *k* by the fourth cycle (depicted in Figure 3l). By stark contrast, UiO‐66─ZrFe/GA experienced a mere 12.8% decline in *k* (as seen in Figure 3l). These results demonstrate that GA‐confined strategy strengthened the formation of Fe(IV)═O.

### 2.4 Mechanism of Electronic Regulation in GA Confinement

**Figure 4:**
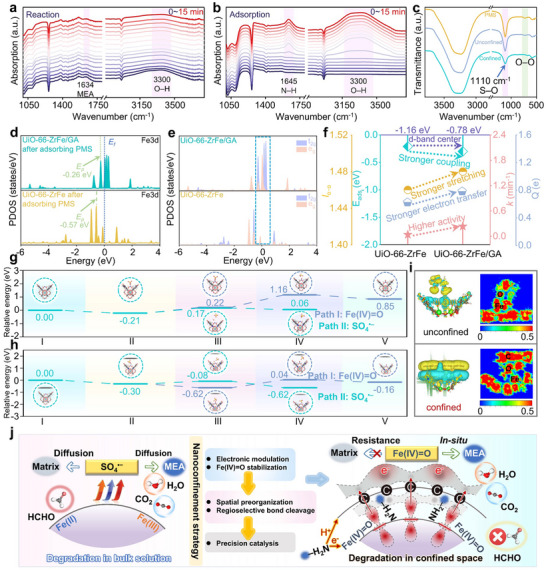
a) In situ ATR‐FTIR spectra during MEA degradation in UiO‐66─ZrFe/GA + PMS system; b) in situ ATR‐FTIR spectra during MEA adsorption; c) solution‐phase FTIR spectra during MEA degradation across catalytic systems; d,e) PDOS for PMS‐adsorbed UiO‐66─ZrFe and UiO‐66─ZrFe/GA; f) correlation between oxidizing capacity, PMS coupling strength, d‐band center, and rate constants; relative energy profiles for SO_4_^•−^ and Fe(IV)═O generation on g) UiO‐66─ZrFe and h) UiO‐66─ZrFe/GA; i) ELF and CDD analyses of PMS activation; j) electron transfer mechanism within the confinement system.

This study employs in situ attenuated total reflection‐Fourier transform infrared (ATR‐FTIR) spectroscopy to probe the evolution of key functional groups during MEA decontamination. The observed continuous attenuation of the hydroxyl (─OH) stretching band exclusively in the degradation system (with PMS, **Figure** **4**a), contrasting its intensification in the absence of PMS (Figure 4b), provides direct molecular‐level evidence for the reaction pathway reconstruction enabled by the confinement strategy. Specifically, the diminishing ─OH signal unambiguously signifies the consumption of MEA's ─OH via Fe(IV)═O‐triggered intramolecular dehydrogenation, while the intensifying band in the PMS‐free system reflects merely reversible, non‐reactive association (e.g., hydrogen bonding). This stark spectral divergence conclusively validates the efficient Fe(IV)═O‐mediated oxidative dehydrogenation. Critically, the irreversible proton uptake by the GA matrix, driving the dehydrogenation reaction to completion, preventing reversibility and manifesting as the persistent decrease in the hydroxyl signature. These observations directly corroborate pathway reconstruction (toward Fe(IV)═O dehydrogenation) and enhanced interfacial mass/charge transfer (proton removal coupled with electron redistribution) as the foundation for accelerated decontamination kinetics. The enhancement of the N─H signal observed in Figure 4b provides further evidence that MEA binds to the Fe sites on catalyst via its ─NH_2_.^[^ ^3^ ^]^ This binding mode is attributed to the higher Fukui index (*f* ^−^) associated with the nitrogen atom of the ─NH_2_ group, signifying that it was the most active electrophilic attack sites, as demonstrated by the computational results presented in Figure S19d (Supporting Information). Liquid FTIR analysis of PMS transformations (Figure 4c) reveals selective attenuation of the S─O/O─O vibrational peak in the UiO‐66─ZrFe/GA + PMS system, providing direct spectroscopic evidence for heterolytic S─O/O─O bond scission—the critical step enabling Fe(IV)═O generation through Fe─O orbital hybridization.

PDOS analysis further reveals distinct Fe‐3d orbital configurations in UiO‐66─ZrFe versus UiO‐66─ZrFe/GA following PMS adsorption. The adsorption strength of intermediates correlates directly with the energy position of the highest electronic peak states (*E*_p_). In Figure 4d, *E*_p_ in PMS‐adsorbed UiO‐66─ZrFe/GA shifts closer to the *E*_f_ than in the UiO‐66─ZrFe system, indicating reduced antibonding state filling and consequently stronger adsorbate bonding. The intensified Fe‐3d (e_g_ and t_2g_) orbital occupancy at *E*_f_ in PMS‐adsorbed UiO‐66─ZrFe/GA originates from interfacial charge redistribution (Figure 4e), where proton‐coupled Fe → C electron transfer during MEA dehydrogenation reduces localized Fe electron density, thereby enhancing PMS → Fe back‐donation into both e_g_ and t_2g_ orbitals. Figure 4f demonstrates that reduced adsorption energy (*E*_ads_) in the UiO‐66─ZrFe/GA + PMS system correlates with enhanced degradation kinetics, while structural and electronic analyses reveal confinement‐driven activation advantages: 1) the elongated O─O bond length (1.48 vs 1.46 Å in UiO‐66─ZrFe) indicates preferential O─O scission capability; 2) greater charge transfer to PMS (0.78 vs 0.62 e^−^) confirms enhanced electron migration, collectively promoting reactive oxygen species liberation and accelerated pollutant degradation. Crucially, the robust correlation among oxidizing capacity, PMS coupling strength, d‐band center position, and pseudo‐first‐order rate constants demonstrates that GA confinement tailors near‐Fermi electronic states at Fe sites, thereby enhancing binding strength and accelerating reaction kinetics.

This study elucidates the GA confinement effect governing SO_4_^•−^/Fe(IV)═O formation pathways during PMS activation—a process fundamentally involving PMS adsorption, metal─PMS electron transfer, and O─O scission.^[^ ^44^ ^]^ The mechanistic pathways proposed in Figure 4g,h were validated through DFT calculations using a cluster model of the Fe‐anchored Zr_6_‐node of UiO‐66. All intermediate structures along both reaction pathways were fully optimized, and their detailed geometries are provided in Figure S20 (Supporting Information). The energy profiles presented in Figure 4g,h are based on the relative energies of these stable intermediates, representing thermodynamic differences between consecutive states. This approach provides robust thermodynamic insight into the origin of the regioselectivity shift induced by GA confinement. Comparative pathway analysis (Figure 4g,h) reveals: For UiO‐66─ZrFe, Path II spontaneously generates SO_4_^•−^ via direct O─O cleavage (−0.11 eV, structure III → IV), while Path I requires proton abstraction (+0.22 eV) followed by rate‐limiting O─O dissociation (+0.94 eV) to form Fe(IV)═O (Structure V). Crucially, GA confinement in UiO‐66─ZrFe/GA restructures activation thermodynamics: 1) enhanced Fe electron distribution reduces PMS adsorption energy to −0.30 eV; 2) path II O─O scission becomes more exergonic (−0.08 eV, Structure III); 3) path I deprotonation barrier plunges to −0.62 eV (Structure III) while rate‐limiting O─O cleavage decreases to +0.66 eV (Structure IV), collectively promoting Fe(IV)═O generation through barrier‐lowered O─O disruption.^[^ ^45^ ^]^ Based on CDD and ELF analyses (Figure 4i), the confined system exhibits significantly enhanced PMS adsorption strength and electron transfer efficiency relative to the unconfined system, thereby directly boosting its activation capability.

Figure 4j schematically elucidates the reaction mechanism of electronic regulation and catalytic pathway transition in UiO‐66─ZrFe/GA + PMS system. In unconfined systems, radical‐mediated MEA degradation occurs in the bulk solution, exhibiting susceptibility to aqueous impurities. Conversely, confining ultrasmall UiO‐66─ZrFe within an asymmetric π‐electron‐rich GA fundamentally shifts the PMS‐activated MEA purification pathway from radical oxidation to an Fe(IV)═O‐dominated mechanism. Critically, lone‐pair electrons on MEA simultaneously induce intramolecular dehydrogenation (releasing H⁺) and reduce Fe(IV)═O. Subsequent proton adsorption by GA synergizes with Fe─PMS orbital hybridization, driving continuous electron transfer from PMS to the carbon matrix and facilitating electron redistribution around Fe active sites. This reconstructed reaction pathway coupled with enhanced interfacial mass transfer accelerates MEA decontamination kinetics and confers exceptional interference resistance, providing a paradigm for designing highly robust advanced oxidation catalytic systems.

### 2.5 Sustainability Assessment and Environmental Applications

**Figure 5:**
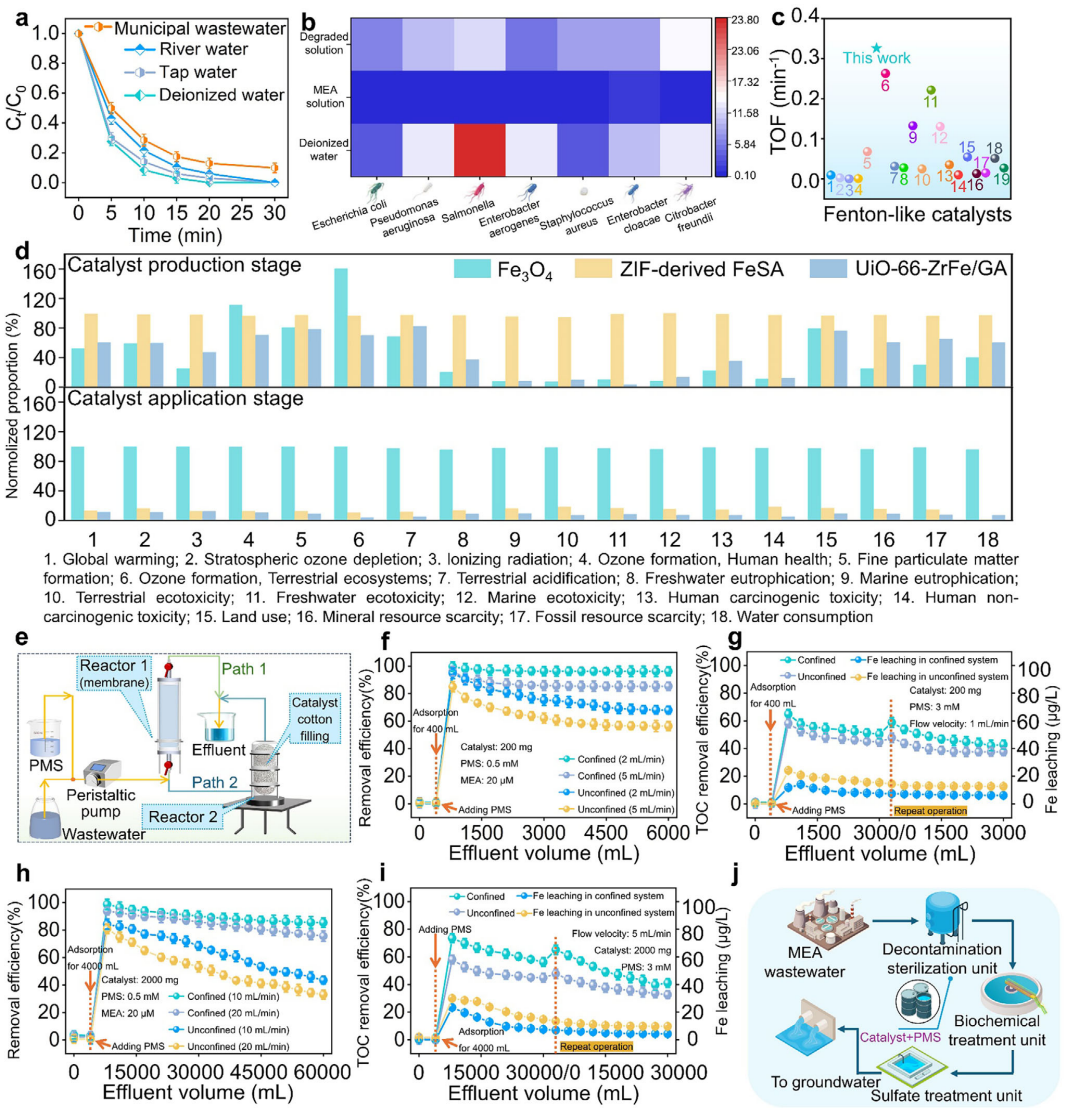
a) Impact of real aqueous matrices on MEA degradation efficiency; b) bacterial growth in different water bodies; c) comparison of turnover frequency (TOF) values with reported Fenton‐like catalysts; d) relative environmental impact of environmentally relevant descriptors for treating MEA using Fe_3_O_4_ + PMS, ZIF‐derived FeSA + PMS, and UiO‐66─ZrFe/GA + PMS systems; e) schematic of continuous‐flow catalytic unit integrated with membrane microreactor or scaled microreactor; f,g) treatment of MEA and iron leaching in membrane microreactor; h,i) treatment of MEA and iron leaching in scaled microreactor; j) proposed industrial wastewater treatment schematic employing UiO‐66─ZrFe/GA as heterogeneous Fenton‐like catalyst. Data points represent the mean values from three independent experimental replicates (*n* = 3), and error bars indicate the standard deviation.

**Figure** **5** shows the catalytic stability test of the UiO‐66─ZrFe/GA + PMS system and assesses its environmental impact. The UiO‐66─ZrFe/GA + PMS system demonstrated excellent oxidative capability in simulated real water matrices (Figure 5a; Figure S21, Supporting Information). To evaluate the generality of the catalytic design principle, the degradation performance of the UiO‐66─ZrFe/GA + PMS system was tested against additional pollutant classes beyond MEA. As shown in Figure S22 (Supporting Information), the system efficiently degraded both the antibiotic sulfamethoxazole and another alkanolamine, diethanolamine, achieving high removal efficiencies within a short reaction time. The successful application to these structurally distinct compounds, featuring different electron‐donating functional groups and molecular architectures, demonstrates that the confinement‐enhanced generation of Fe(IV)═O and the associated proton–electron coregulation mechanism are not specific to MEA. This broad reactivity underscores the potential of our engineered catalyst as a versatile platform for the treatment of diverse organic contaminants, thereby substantiating the claim of a generalized design principle.

Bacterial proliferation assays (Figure 5b; Table S5, Supporting Information) revealed seven strains (including *Escherichia coli*, *Enterobacter cloacae*) in pure water and MEA‐degraded solution after 48 h incubation. Notably, nonpigmented strains (e.g., *Pseudomonas aeruginosa*, *Staphylococcus aureus*) persisted in MEA solution controls, confirming the system's effective detoxification.^[^ ^46^ ^]^ To further substantiate the environmental compatibility of the treatment process, the acute toxicity of the solutions before and after reaction was quantitatively evaluated using a luminescent bacteria (*Vibrio fischeri*) inhibition assay. The test was conducted by exposing the bacteria to the original MEA solution and the solution sampled after 2.5 h of treatment with the UiO‐66─ZrFe/GA + PMS system. As shown in Figure S23 (Supporting Information), the inhibition of luminescence decreased dramatically from over 80% in the initial MEA solution to below 20% after treatment. This marked reduction in acute toxicity occurred despite the presence of intermediate products such as formic and acetic acids, which are common, less‐toxic degradation intermediates in AOPs that are ultimately mineralizable to CO_2_ and H_2_O. This result, consistent with the earlier bacterial proliferation assay (Figure 5b), provides direct experimental evidence for the effective detoxification achieved by our catalytic system.UiO‐66─ZrFe/GA exhibited superior catalytic efficiency with a turnover frequency (TOF) value (0.326 min^−1^) exceeding most reported Fenton‐like catalysts (Figure 5c; Table S6, Supporting Information). Life cycle assessment (LCA) quantified environmental impacts of UiO‐66─ZrFe/GA + PMS relative to conventional Fe_3_O_4_ + PMS and ZIF‐derived FeSA + PMS systems. Analysis covered catalyst synthesis and pollutant degradation phases using 18 descriptors (lab‐scale data; Figure S24, Supporting Information). Fe_3_O_4_ preparation showed lower impacts due to simpler synthesis (Figure 5d; Text S12, Supporting Information), while UiO‐66─ZrFe/GA synthesis proved more eco‐efficient than ZIF‐derived FeSA. Crucially, UiO‐66─ZrFe/GA's higher intrinsic activity reduced catalyst/PMS requirements and energy consumption during MEA degradation, yielding lower operational impacts than ZIF‐derived FeSA. This advantage, coupled with anticipated scale‐driven resource optimization, positions UiO‐66─ZrFe/GA as an environmentally superior technology.

The superior properties of UiO‐66─ZrFe/GA motivated device‐level implementation for practical wastewater treatment. Two continuous‐flow microreactors (Figure 5e; Figure S25 and Text S13, Supporting Information) evaluated long‐term stability using simulated and actual wastewater. In the membrane microreactor, UiO‐66─ZrFe/GA completely remediated 6 L MEA wastewater at 2 mL min^−1^ but exhibited declining efficiency at 5 mL min^−1^ due to pollutant overloading (Figure 5f). UiO‐66─ZrFe showed inferior stability under high flow rates. UiO‐66─ZrFe/GA also demonstrated enhanced TOC removal versus UiO‐66─ZrFe (Figure 5g), maintaining ≈50% mineralization even during effluent recirculation at 1 mL min^−1^. The scaled‐up reactor (Figure 5h,i) achieved >80% MEA removal at 10─20 mL min^−1^ while processing 60 L wastewater, confirming catalytic robustness. This confined system attained 38.8–41.2% TOC and 81.4–88.4% chemical oxygen demand removal (Table S7, Supporting Information). Fe leaching remained below 100 µg L^−1^ (Figure 5i), meeting the GB 5749‐2022 drinking water standard (300 µg L^−1^). The scalable reactor design demonstrates practical potential for industrial MEA treatment (Figure 5j), establishing a sustainable water purification platform.

Figure S19a (Supporting Information) depicts the MEA molecular structure, while Figure S19b (Supporting Information) shows the van der Waals surface electrostatic potential (ESP) distribution. Red regions (negative ESP) denote electron‐rich areas; blue regions (positive ESP) indicate electron‐deficient zones. Figure S19c (Supporting Information) displays the highest occupied molecular orbital (HOMO) distribution, identifying reactive sites. HOMO electrons are susceptible to electrophilic attack; in MEA, the HOMO concentrates on the nitrogen atom, enhancing its reactivity.^[^ ^15^ ^]^ MEA degradation products in UiO‐66─ZrFe + PMS and UiO‐66─ZrFe/GA + PMS systems are shown in Figure S19e–l (Supporting Information). Confined microenvironments enabled near‐complete MEA mineralization, reducing formaldehyde yield by 86.4% (Figure S19f,j, Supporting Information). Catalysis converted MEA into valuable carbon sources (formic acid: 11.8%; acetic acid: 24.1%; Figure S19j, Supporting Information), suggesting compatibility with biotreatment. Ecological structure activity relationships (ECOSAR) and toxicity estimation software tool and modeling predicted intermediate ecotoxicity (Figure S26, Text S14, Tables S8 and S9, Supporting Information): most products exhibited substantially lower toxicity than MEA, except formaldehyde. Furthermore, the UiO‐66─ZrFe/GA + PMS system achieved exceptional degradation, enabled by synergistic GA layer size‐exclusion and hollow microenvironment confinement effects that enhanced Fe(IV)═O selectivity (Figure S27a,b, Supporting Information). The GA layer excluded macromolecules (e.g., humic acid; Figure S28a,b, Supporting Information). Unlike radicals, Fe(IV)═O resisted ionic interference. Economic analysis (Text S15, Supporting Information) yielded an EE/*O*_total_ of 3.55 kWh m^−3^ ($0.47 m^−3^), outperforming prior advanced oxidation systems.^[^ ^47^ ^]^ Strategic manipulation of reaction microenvironments via nanoconfinement offers valuable insights for developing efficient catalytic technologies.

### 2.6 Perspectives on Industrial Applicability and Scalability

While the lab‐scale performance and preliminary LCA are promising, a realistic assessment of industrial applicability must address key practical considerations. A comparative cost analysis in Tables S10 and S11 (Supporting Information) indicates that the synthesis of UiO‐66─ZrFe/GA is currently more expensive than conventional Fe‐based catalysts like Fe_3_O_4_, primarily due to the cost of organic linkers and solvents. However, it remains substantially more cost‐effective than advanced Fe‐based catalysts such as ZIF‐derived FeSA, while offering superior environmental benefits as demonstrated in our LCA. To evaluate practical stability, we conducted an extended 20 days test using real carbon capture wastewater, where the catalyst maintained a high MEA removal efficiency of 78.8%, demonstrating robust performance in a complex industrial matrix (Figure S29, Supporting Information). Potential pathways for cost reduction exist, including solvent recycling and the adoption of continuous‐flow synthesis methods, which have shown promise for the scalable production of other MOFs. The long‐term stability of the catalyst under real wastewater conditions over periods of months remains a critical challenge, though the negligible metal leaching and robust performance in our extended lab‐scale operation provide a positive initial indicator. Adapting such established scale‐up techniques, coupled with the inherent processability of the GA support which effectively addresses the powder handling issues common to nanoscale MOFs, presents a feasible, though nontrivial, path toward larger‐scale manufacturing. This discussion underscores that while engineering challenges lie ahead, the fundamental design principle offers a compelling direction for developing more efficient and selective advanced oxidation technologies.

## 3 Conclusion

This work establishes an asymmetric π‐electron‐enriched GA confinement strategy that fundamentally reconstructs PMS activation pathways by synergistically integrating spatial constraints with the engineering of interfacial electron/proton flux. The radially divergent electron gradient field generated by lattice distortion‐induced π‐orbital rehybridization forcibly polarizes charge transfer, enabling: 1) preferential heterolytic O─O scission for targeted Fe(IV)═O generation; 2) a unique proton–electron coregulation cycle where MEA's ─NH_2_ groups reduce Fe(IV)═O while triggering intramolecular C_α_─H dehydrogenation; 3) GA‐mediated proton capture inducing π‐system symmetry breaking, synergizing with Fe (3d)─PMS (2p) hybridization to drive directional electron transfer from PMS antibonding orbitals. This study demonstrates that the coupling of molecular preorganization within confined spaces with this self‐sustaining proton–electron cycle is the key innovation, enabling precise regioselectivity. This concerted mechanism yielded a high‐performance system, achieving a 4.85‐fold enhancement in MEA degradation kinetics, 90.3% PMS utilization efficiency, and a leap in TOC mineralization from 29.9% to 74.7%, while effectively suppressing genotoxic formaldehyde byproduct formation to 2.3%. The catalyst demonstrated exceptional practical robustness, maintaining high degradation efficiency (≈80% over 20 days in real wastewater and over 85% in a 60 L continuous‐flow test) with negligible iron leaching (<100 µg L^−1^), and performance remained uncompromised under challenging environmental interferents. This confinement eliminates radical‐mediated constraints, while electronic reprogramming overcomes mass‐transfer limitations and parasitic quenching. The system thus presents an advanced AOP platform that synchronizes nanoconfinement engineering with atomic‐level pathway control, providing a generalized design principle based on orchestrating coupled spatial and electronic effects for selective oxidation.

## Conflict of Interest

The authors declare no conflict of interest.

## Supporting information



Supporting Information

## Data Availability

The data that support the findings of this study are available in the supplementary material of this article.
