# Supplementary material for: Nanoconfinement‐Steered Molecular Preorganization Enables Efficient Monoethanolamine Degradation through Electronically Modulated High‐Valent Iron–Oxo Pathways
Source: Adv Sci (Weinh). 2025 Nov 25;13(8):e14979. doi: 10.1002/advs.202514979 (PMC12884791; doi:10.1002/advs.202514979)
Supplement: Supplementary file 1 — Supporting Information [file ADVS-13-e14979-s001.pdf]

Supporting Information

Nanoconfinement-Steered Molecular Pre-Organization Enables Efficient Monoethanolamine Degradation through Electronically Modulated High-Valent Iron-Oxo Pathways

*Lin Zhang, Qin Dai, Xinyue Chen, Hang Yuan, Lei Xing, Zhimo Fang, Xiuze Li, Xiangke Wang, Lihui Zhang, Junping Zhao, Qiangwei Li, and Lidong Wang**

L. Zhang (*Lin Zhang*), Q. Dai, X. Chen, H. Yuan, L. Xing, X. Li, X. Wang, Q. Li, L. Wang

MOE Key Laboratory of Resources and Environmental Systems Optimization, College of Environmental Science and Engineering, North China Electric Power University, Beijing, 102206, P. R. China

E-mail: wld@ncepu.edu.cn (Lidong Wang)

Z. Fang

National Engineering Research Center of New Energy Power Generation, North China Electric Power University, Beijing 102206, P. R. China

L. Zhang (*Lihui Zhang*), J. Zhao

Institute of Energy Resources, Hebei Academy of Sciences, Shijiazhuang, 050081, P. R. China

Contents

Text S1. Chemicals and reagents.
Text S2. Catalyst preparation.
Text S3. Degradation performance evaluation.
Text S4. Characterization methods.
Text S5. XAFS measurements.
Text S6. Calculation of reaction rate.
Text S7. Evaluation of PMS utilization rate.
Text S8. Calculation details of the turnover frequency.
Text S9. Computational methods.
Text S10. Effects of catalyst dosage, PMS concentration, and MEA concentration.
Text S11. Detection of Fe(IV)=O.
Text S12. Methods, systems boundaries and inventory of life cycle assessment.
Text S13. Continuous flow experiment for water treatment.
Text S14. Toxicity assessment.
Text S15. Economic analysis.
Text S16. Statistical analysis.
Figure S1. Schematic illustration of the synthetic procedure of UiO-66-ZrFe/GA.
Figure S2. TEM images of (a) GA and (b) UiO-66-ZrFe.
Figure S3. (a) N_2 adsorption/desorption isotherms and (b) pore size distribution of UiO-66-ZrFe/GA.
Figure S4. Reproducibility of Zr 3d XPS spectra.
Figure S5. The removal of MEA by catalysts or PMS alone.
Figure S6. Effects of (a) catalyst dosage, (b) PMS concentrations, and (c) MEA concentrations; (d) the *k* under various conditions.
Figure S7. Effects of initial pH.
Figure S8. The essential role of Fe(II) validated by ferrozine tests.
Figure S9. The XRD of fresh and used UiO-66-ZrFe/GA.
Figure S10. The concentrations of leached Fe during the consecutive cycles.
Figure S11. The removal efficiency of MEA in homogeneous systems. Reaction conditions: $[Fe^{2+}] = [Fe^{3+}] =$ 0.01 mg/L.
Figure S12. (a) The influences of various scavengers in UiO-66-ZrFe+PMS system and (b) the influences of various scavengers on *k*.
Figure S13. (a) The influences of various scavengers in UiO-66-ZrFe/GA+PMS system and (b) the influences of various scavengers on *k*.
Figure S14. EPR spectra of $SO_4^{\bullet-}$ and $^{\bullet}OH$ in unconfined UiO-66-ZrFe+PMS system and confined UiO-66-ZrFe/GA+PMS system.
Figure S15. (a) The analysis of integrated peak areas showing the decay of PMS bands and the formation of a steady-state Fe(IV)=O concentration; (b) isotopic shift of the Fe(IV)=O vibration from 838 cm^{-1} in $H_2{}^{16}O$ to 827 cm^{-1} in $H_2{}^{18}O$, providing definitive evidence for the Fe=O bond assignment. These results collectively demonstrate the dynamic steady-state behavior of the Fe(IV)=O intermediate within the confined catalytic microenvironment.
Figure S16. PMSO consumption and $PMSO_2$ generation in UiO-66-ZrFe/GA+PMS system.
Figure S17. Competition experiment demonstrating the dominance of the Fe(IV)=O pathway.
Figure S18. Degradation efficiency and *k* of (a) UiO-66-ZrFe/GA and (b) UiO-66-ZrFe in consecutive cycles.
Figure S19. (a) Chemical structure of MEA; (b) ESP; (c) HOMO; (d) Fukui index; (e, f) carbon-containing products and (g, h) nitrogen-containing products in UiO-66-ZrFe+PMS system; (i, j) carbon-containing products and (k, l) nitrogen-containing products in UiO-66-ZrFe/GA+PMS system.

Figure S20. Optimized geometries of reaction intermediates for PMS activation on UiO-66-ZrFe and UiO-66-ZrFe/GA.
Figure S21. Effects of actual water samples in UiO-66-ZrFe+PMS system.
Figure S22. Evaluation of catalytic generality across different pollutant classes. Reaction conditions: $[Pollutant]_0$ = 20 mg/L, [PMS] = 2 mM, catalyst loading = 0.2 g/L, unadjusted pH.
Figure S23. Acute toxicity assessment of the treated solution using a luminescent bacteria (Vibrio fischeri) bioassay.
Figure S24. System boundaries for the processes studied in this work.
Figure S25. Photographs of the operation of the continuous-flow experiment conducted with (a) an enlarged microreactor and (b) a membrane reactor.
Figure S26. (a) The acute and (b) chronic toxicity obtained from ECOSAR program; (c)–(f) toxicity assessment based on TEST program.
Figure S27. Effects of ions in UiO-66-ZrFe+PMS and UiO-66-ZrFe/GA+PMS systems. The reaction conditions: $[MEA]_0$ = 20 mg/L, [PMS] = 2 mM, [catalyst] = 0.2 g/L, $[ion]_0$ = 3 mM, natural pH.
Figure S28. Effects of humic acid in UiO-66-ZrFe+PMS and UiO-66-ZrFe/GA+PMS systems, $[HA]_0$: 5 mg/L and 10 mg/L.
Figure S29. Evaluation of catalytic performance using real industrial waste streams. (a) Photograph of the actual carbon capture plant and the collected spent MEA wastewater used in this study; (b) long-term stability test: MEA removal efficiency by the UiO-66-ZrFe/GA+PMS system over 20 days of continuous operation with the real spent MEA wastewater. Reaction conditions: $[MEA]_0$ = 2 g/L, [PMS] = 200 mM, catalyst loading = 20 g/L.
Table S1. The HPLC methods and conditions for persistent organic contaminants.
Table S2. BET-specific surface area, pore volume, and pore size of UiO-66-ZrFe/GA.
Table S3. EXAFS fitting parameters at the Fe K-edge for various samples (S_0^2=0.84).
Table S4. The element contents of fresh and used UiO-66-ZrFe/GA catalysts from ICP.
Table S5. Salmonella chromogenic medium in 37°C train 48 h.
Table S6. Catalytic activity comparison of UiO-66-ZrFe/GA with currently reported Fenton-like catalysts in PMS-mediated pollutant removal.
Table S7. Performance of the UiO-66-ZrFe/GA+PMS system applied in the enlarged microreactor device for the continuous treatment of actual municipal wastewater samples (water samples were filtered through 0.45 μm membranes before treatment, and the recorded after-treatment data were the parameters after repeated operations).
Table S8. Estimation of acute and chronic toxicity of MEA and its transformation products to fish, daphnid, and green algae using the ECOSAR program.
Table S9. Estimation of the Oral rat LD50, mutagenicity, bioaccumulation factor, and developmental toxicity for MEA and its transformation products using the TEST program.
Table S10. Input materials and energy required in the catalyst production stage to degrade 1 kg MEA through Fe_3O_4/PMS, ZIF-derived FeSA/PMS and UiO-66-ZrFe/GA+PMS processes.
Table S11. Comparative cost analysis of three catalysts

Texts

Text S1. Chemicals and reagents.

All chemicals and reagents were at least analytical grade and used as received without any treatment. Monolayer graphene oxide (GO) powder sample (>99 wt%, 0.5-3.0 nm in thickness) was purchased from Suzhou Tan Feng Graphene Technology Co., Ltd., China. Zirconium tetrachloride ($ZrCl_4$) was purchased from Meryer Biochemical Technology Co., Ltd, China. 2-Aminoterephthalic acid (H_2ATA) was purchased from Macklin Biochemical Technology Co., Ltd, China. Ferric chloride ($FeCl_3 \cdot 6H_2O$), ferrous sulfate ($FeSO_4 \cdot 7H_2O$), 2,2,6,6-tetramethyl-1-piperidinyloxy (TEMP), phenylmethyl sulfoxide (PMSO), methyl phenyl sulfone ($PMSO_2$), ethanol (EtOH), methanol (MeOH), acetonitrile, sodium chloride (NaCl), sodium sulfate (Na_2SO_4), sodium hydroxide (NaOH), magnesium sulfate ($MgSO_4$), monoethanolamine (MEA), $KHSO_5 \cdot 0.5KHSO_4 \cdot 0.5K_2SO_4$ (PMS), and humic acid (HA) were purchased from Aladdin Industrial Corporation, China. Ethylenediamine (EDA), N, N-dimethylformamide (DMF), polyvinylpyrrolidone (PVP), sodium nitrate ($NaNO_3$), hydrochloric acid (HCl), and potassium iodide (KI) were purchased from Sinopharm Chemical Reagent Co. Ltd., China. Tert-butanol (TBA) was obtained from the Chengdu Kelong reagent factory.

Text S2. Catalyst preparation.

GA was synthesized via hydrothermal methodology.[1] Specifically, 0.03 mL ethylenediamine (EDA) and 30 mg commercial GO were dispersed in 10 mL deionized water. The mixture underwent ultrasonication for 30 min to ensure homogeneity prior to hydrothermal reaction (180°C, 5 h) in a Teflon-lined autoclave. The resultant hydrogel was cooled to ambient temperature, immersed in 15% ethanol for 24 h stabilization, and vacuum-dried at 60°C for 24 h.

For UiO-66-Zr synthesis, 466 mg $ZrCl_4$ and 362 mg H_2ATA were dissolved in 40 mL N,N-dimethylformamide (DMF) under vigorous stirring for 60 min. The solution was subsequently reacted at 150°C for 24 h. The crystalline product was collected by centrifugation, washed sequentially with deionized water and ethanol (3 cycles each), and dried at 80°C for 24 h.

The UiO-66-ZrFe/GA composite was fabricated by initially dispersing 30 mg GA in 40 mL DMF containing 233 mg $ZrCl_4$, 270 mg $FeCl_3 \cdot 6H_2O$, 362 mg H_2ATA, and 200 mg polyvinylpyrrolidone (PVP). This dispersion underwent continuous stirring for 60 minutes followed by solvothermal treatment at 150°C for 24 hours in a Teflon-lined autoclave. Subsequent purification involved sequential washing with ethanol and deionized water, concluding with vacuum drying at 60°C for 24 hours. Control samples comprised: UiO-66-ZrFe (synthesized identically without GA addition), and a physically blended GA+UiO-66-ZrFe composite (1:1 mass ratio).

Text S3. Degradation performance evaluation.

Quantification of MEA and potential intermediates was performed using ion chromatography (IC) with a CIC-D100 instrument (Qingdao Shenghan, China) under cationic conditions. MEA degradation experiments were conducted at ambient temperature under natural initial pH conditions. To quantify residual MEA concentrations, reaction aliquots were periodically extracted during the degradation process. Radical quenching studies employed methanol (MeOH, 200 mM), tert-butanol (TBA, 200 mM), 2,2,6,6-tetramethyl-1-piperidinyloxy (TEMP, 10 mM), and dimethyl sulfoxide (DMSO, 10 mM) as selective scavengers for $SO_4^{\bullet-}$, $^{\bullet}OH$, $^1O_2$, and Fe(IV)=O species respectively. Post-reaction, the UiO-66-ZrFe/GA catalyst was recovered via centrifugation for consecutive cycling tests. The recovered material subsequently underwent rigorous purification comprising three sequential washes with deionized water and ethanol, followed by vacuum drying at 60°C for 12 h.

Text S4. Characterization methods.

The crystal phase and morphology of the as-prepared catalysts were tested by X-ray diffractometer (XRD, Shimadzu XRD-6100), scanning electron microscope (SEM, JEOL JSM-7800F Prime), and transmission electron microscope (TEM, TALOS F200X). The X-ray absorption fine structure (XAFS) spectra data was collected, and the detailed methods were provided in Text S5. The concentration of persistent organic contaminants was determined with high-performance liquid chromatography (HPLC, Agilent, 1260-Infinity). The details of analytical methods and conditions are summarized in Table S1. The elemental composition and concentration were quantified by an Inductively Coupled Plasma Mass Spectrometry (ICP-MS, Agilent 7700). Electron paramagnetic resonance (EPR) spectra were recorded on a JES-FA200 spectrometer. The total organic carbon (TOC) of reaction solutions was determined by a TOC analyzer (Multi N/C 3100, Analyticjena, Germany). Raman spectroscopy (Renishaw InVia Raman microscope) was obtained with a He–Ne laser (λ = 532 nm) as the excitation source. The ^{18}O isotope labeling experiments were carried out in 5.0 mL $H_2{}^{18}O$. The $^{16}O/^{18}O$ isotope-labeled $PMSO_2$ was identified by High-Performance Liquid Chromatography Quadrupole Time-of-Flight Tandem Mass Spectrometry (HPLC−QTOF−MS, G6500, Agilent, USA) with negative (−EIS) mode.

Text S5. XAFS measurements.

The XAFS data were collected at the BL1W1B station in the Beijing Synchrotron Radiation Facility (BSRF, operated at 2.5 GeV with a maximum current of 250 mA). The data of the samples were collected at room temperature (Fe K-edge in fluorescence excitation mode using a Lytle detector). All samples were pelletized as disks of 13 mm diameter with 1 mm thickness using PVDF powder as a binder.

The extended XAFS (EXAFS) spectra were obtained by subtracting the post-edge background from the overall absorption and then normalizing with respect to the edge-jump step. Subsequently, the χ(k) data of Fourier transformed to real (*R*) space using hanning windows (dk = 1.0 $Å^{-1}$) to separate the EXAFS contributions from different coordination shells. To obtain the quantitative structural parameters around central atoms, least-squares curve parameter fitting was performed using the ARTEMIS module of the IFEFFIT software packages.

The following EXAFS equation was used:

$$\chi(k)=\sum_j \frac{N_j S_o^2 F_j(k)}{k R_j^2}\exp[-2k^2\sigma_j^2]\exp[\frac{-2R_j}{\lambda(k)}]\sin[2kR_j+\phi_j(k)] \quad \text{(S1)}$$

$S_0{}^2$ is the amplitude reduction factor, $F_j(k)$ is the effective curved-wave backscattering amplitude, N_j is the number of neighbors in the jth atomic shell, R_j is the distance between the X-ray absorbing central atom and the atoms in the jth atomic shell (backscatter), λ is the mean free path in Å, $\phi_j(k)$ is the phase shift (including the phase shift for each shell and the total central atom phase shift), σ_j is the Debye-Waller parameter of the jth atomic shell (variation of distances around the average R_j). The functions $F_j(k)$, λ, and $\phi_j(k)$ were calculated with the ab initio code FEFF8.2. The additional details for EXAFS simulations are given below.

The coordination numbers of model samples were fixed as the nominal values. The obtained $S_0{}^2$ was fixed in the subsequent fitting. While the internal atomic distances *R*, Debye-Waller factor σ^2, and the edge-energy shift ΔE_0 were allowed to run freely.

Text S6. Calculation of reaction rate.

Equation S2: The equation for the kinetic rate constant of the first order.[2]

$$\ln(C_0/C_t) = kt \quad \text{(S2)}$$

Where C_0 and C_t represent the concentrations of MEA at initial and specific time (t), *k* is the rate constant

(min^{-1}), and t is the reaction time (min).

Text S7. Evaluation of PMS utilization rate.

Residual PMS concentration was determined by extracting 0.5 mL of reaction mixture into 4.5 mL potassium iodide solution (1.11 g/L KI).[3] The combined solution underwent vigorous vortex mixing for 30 min to ensure stoichiometric conversion of HSO_5^-, to I_3^- via iodide oxidation, as described by Equation S3–S4. Subsequent spectrophotometric quantification of I_3^- concentration, which correlates linearly with initial PMS concentration, was performed at 352 nm using Ultraviolet and Visible Spectrophotometer.

$$2I^- + HSO_5^- + 2H^+ \rightarrow HSO_4^- + I_2 + H_2O \quad (S3)$$

$$I_2 + I^- \rightarrow I_3^- \quad (S4)$$

Text S8. Calculation details of the turnover frequency.

The turnover frequency (TOF) of each metal site, which represents the intrinsic activity of the catalyst could be calculated as follows:

$$\text{TOF } [\text{min}^{-1}] = \frac{moles\ of\ reactant\ converted}{moles\ of\ active\ sites \times reaction\ time} = \frac{\Delta n(pollutant)}{n_{metal} \times t} = \frac{\Delta n(pollutant) \times M_{metal}}{m_0 \times \omega_{metal} \times t} \quad (S5)$$

where $\Delta n(pollutant)$ is the moles of pollutants converted, M_{metal} is the atomic weight of metal, m_0 is the mass dosing of catalysts, ω_{metal} is the mass concentration of metal in the catalysts, t is the reaction time.

Text S9. Computational methods.

We selected the Projector-Augmented-Wave (PAW) potentials with Perdew-Burke-Ernzerhof (PBE) of exchange-correlation interactions as performed in the Vienna Ab initio Simulation Package (VASP 5.4.1) code to describe the ion nuclei and took the valence electrons into account using a plane wave basis set with kinetic energy cutoff to 400 eV. The electron energies were deemed to be self-consistent when the energy change was less than 10^{-4} eV. The geometric optimization was believed to be convergent when the energy change was less than 0.05 eV Å^{-1}. The vacuum spacing was 15 Å in the direction perpendicular to the structure plane. The integration in the Brillouin zone was sampled using 2×2×1 Monkhorst-Pack k-point sampling for one structure. The climbing image nudged elastic band (NEB) method was utilized to locate the minimum energy pathways (MEPs) from an initial state (IS) to its final state, and the transition state (TS) was localized using the climbing image method and verified with a single imaginary frequency. The adsorption energy Eads is defined as follows:

$$E_{ads} = E_{PMS/catalyst} - (E_{PMS} + E_{catalyst}) \quad (S6)$$

where $E_{PMS/catalyst}$, E_{PMS}, and $E_{catalyst}$ are the total energies of the PMS/catalyst system, the isolated PMS molecule, and the catalyst in the same slab, respectively.

The Gaussian 09 program was used to analyze the MEA molecule and obtain the HOMO distribution through the B3LYP/6-311+G (2d, 2p) method. The Fukui index was calculated by using Multiwfn software.

Text S10. Effects of catalyst dosage, PMS concentration, and MEA concentration.

Figure S6a and S6d shows the MEA degradation efficiency of 88%, 100%, and 100%, corresponding to k of 0.134, 0.234, and 0.399 min^{-1}. Under the same UiO-66-ZrFe/GA concentration, the degradation efficiency of MEA was also associated with the PMS dosage. The degradation efficiencies were 92.2%, 100%, and 100%, corresponding to k of 0.157, 0.234, and 0.335 min^{-1} (Figure S6b and S6d), respectively. Considering the economic benefit, the optimal concentrations of UiO-66-ZrFe/GA and PMS were 0.2 g/L and 2 mmol/L, respectively. With the increase in MEA concentration (from 10 to 30 mg/L) (Figure S6c), the degradation

efficiency decreased to 91%. As pollutant concentration enhanced, the reactive oxygen species was insufficient.[4] Excessive MEA might occupy the active sites of UiO-66-ZrFe/GA, causing decreased performance.[5]

Text S11. Detection of Fe(IV)=O.

^{18}O isotope-labeled experiments were performed in 10 mL $H_2{}^{18}O$ matrix under preset conditions. The HPLC-QTOF-MS/MS analysis of PMSO, $PMS^{16}O^{16}O$, and $PMS^{16}O^{18}O$ were measured by ultra-performance liquid chromatography combined with quadrupole time-of-flight mass spectrometry (UPLCQ-TOF-MS/MS, Agilent 1290 Infinity II UPLC with Agilent G6545 Q-TOF) under the following conditions. Separation was performed with a BEH C18 column (100 mm × 2.1 mm i.d., 1.7 μm; Waters, Milford, USA) and eluted with gradient solvent from A: B (95: 5) to A: B (5: 95) at a flow rate of 0.20 mL min^{-1}, where A is aqueous formic acid (0.1% (v/v)) and B is acetonitrile. The column was maintained at 40 °C. Accurate MS/MS patterns and oxidation products were analyzed in a molecular ion scanning mode (m/z 20 to 1000) in positive electrospray ionization (ESI) modes. The molecular formula of identified products was proposed based on experimental and theoretical m/z values.

Text S12. Methods, systems boundaries and inventory of life cycle assessment.

Goal and scope. The assessments aimed to determine whether the UiO-66-ZrFe/GA and the catalytic system coupled with PMS results in reduced carbon emissions, energy consumption, and other environmental impacts when applied to water treatment as compared to typical Fe-based catalysts.

Scenario description, system boundaries and life cycle inventory. Three scenarios (Fe_3O_4+PMS process, ZIF-derived FeSA+PMS process, and UiO-66-ZrFe/GA+PMS process) were considered in this study (Figure 5d). The functional unit for all processes was calibrated for the treatment of 1 kg MEA. Figure S24 shows the system boundaries including (i) catalyst production stage and (ii) catalyst application stage. By contrast, the detailed inventory data of the Fe_3O_4+PMS process, ZIF-derived FeSA+PMS process are based on the literature.[6]

Assessment methods. The LCA was simulated using SimaPro 93. Based on the above data, ReCiPe method (v1.07) was applied to assess environmental impacts. In this study, the environmental impacts were classified into 18 midpoint indicators, including global warming, stratospheric ozone depletion, ozone formation for human health, ozone formation for terrestrial ecosystems, fine particulate matter formation, terrestrial acidification, freshwater eutrophication, marine eutrophication, terrestrial ecotoxicity, freshwater ecotoxicity, marine ecotoxicity, human carcinogenic toxicity, human non-carcinogenic toxicity, mineral resource scarcity, fossil resource scarcity and water consumption, which were obtained at the midpoint level.

Text S13. Continuous flow experiment for water treatment.

In the continuous flow setup of reactor 1, 200 mg of the synthesized UiO-66-ZrFe/GA was dispersed uniformly in 200 mL of deionized water and subjected to ultrasonic agitation for 30 minutes to ensure thorough dispersion. Subsequently, UiO-66-ZrFe/GA membrane was fabricated by passing this homogenized suspension through a cellulose acetate membrane under continuous vacuum filtration. Following filtration, the membrane was removed, allowed to air-dry for 1 hour, and then further dried in an oven at 40°C for an additional hour to ensure complete moisture removal.

High-density cotton balls are light weight and low-cost, as well as possessing well-developed pores, high strength, good corrosion resistance, good flexibility, and strong resistance to shock loads, making them an ideal carrier for supporting UiO-66-ZrFe/GA. In the continuous flow setup of reactor 2, the cotton balls (φ = 4 cm) were immersed in UiO-66-ZrFe/GA aqueous solution (2 mg/mL) and repeatedly squeezed for 5 min, and then vacuum dried at 180°C for 2 h after adequate adsorption to obtain the cotton balls with stable loading

of UiO-66-ZrFe/GA. The average mass of catalyst loaded per cotton ball was 200 mg, and the reactor was filled with 10 cotton balls loaded with UiO-66-ZrFe/GA for each run.

Text S14. Toxicity assessment.

During the MEA degradation, various toxic intermediates could be formed. The ecotoxicity of MEA and its products was evaluated by ECOSAR and TEST programs through the Quantitative Structure and Activity Relationship (QSAR) method.[7] Table S8, Figure S26a, and S26b display the toxicity of intermediates for fish, daphnid, and green algae calculated by the ECOSAR program. The LC50 and EC50 values for some of the byproducts were generally higher than those of MEA. The intermediates showed a similar toxicity order in the aspects of acute and chronic toxicities. However, the intermediate (such as formaldehyde) had stronger toxicities than MEA. The oral rat LD50, mutagenicity, bioaccumulation factor, and developmental toxicity were evaluated based on the TEST program (Table S9). In Figure S26c, the LD50 values of some intermediates were higher than MEA, indicating that they were comparatively less toxic than parent MEA for oral rats. Figure S26d shows the mutagenicity results, and all products except formaldehyde were lower than MEA. As shown in Figure S26e, these products had lower bioaccumulation factors than MEA. They were less easily enriched in organisms than MEA. Developmental toxicity refers to the ability to influence nucleic acid's translation and expression, as well as individual growth. Figure S26f indicated that the predicted developmental toxicity of some compounds was lower than the original MEA. Different products had respective toxicity, but they could eventually be adequately mineralized. Therefore, the UiO-66-ZrFe/GA+PMS system is advisable for degrading the organics to decrease the ecotoxicity. It is meaningful for environmental and ecological assessment.

Text S15. Economic analysis.

The electrical energy per order (EE/O) has been used as a popular approach for evaluating the energy and cost of reaction systems.[8] It is defined as electrical required to reduce the content of a specific pollutant by one order of magnitude.[8a] This method often involves consumed electrical energy and chemicals, where the catalyst is usually assumed to be reusable, so it is not considered a consumable in the system.[8] Herein, the economic evaluation of the optimal UiO-66-ZrFe/GA+PMS system was performed based on the EE/O_{total} using the following Equation (S7), (S8), and (S9).[9] Due to the lack of input of external energy, such as light[10] and ultrasonic energy[11] in the UiO-66-ZrFe/GA+PMS system, the external energy input was zero. Therefore, the value of EE/O_{total} would be converted from PMS/O.[9] According to the literature,[12] the non-house electrical energy cost and PMS were estimated to be 0.1319 \$/kWh and 1.30 \$/kg. Thus, the electrical energy cost of PMS was calculated as 9.85 kWh/kg.[9] Then, based on the results of the Figure 3a and 3e, 97% of MEA was removed, and 89.3% of PMS was consumed within 15 min. The calculated EE/O_{total} value of the UiO-66-ZrFe/GA+PMS system was 3.55 kWh/m^3, and the corresponding cost was 0.47 \$/m^3. The cost was almost lower than that of advanced oxidation processes in previously reported literature.[11] Based on the analysis, our UiO-66-ZrFe/GA+PMS system presented a much more promising application potential in cost and environmental friendliness.

$$E/O_{Total} = EE/O_{Energy} + EE/O_{Chemicals} = EE/O_{Chemicals} \quad \text{(S7)}$$

$$E/O_{Chemicals} = PMS/O = \frac{[PMS]_0 - [PMS]_f}{lo\left(\frac{C_i}{C_f}\right)} (mgPMS/L) \quad \text{(S8)}$$

$$ln\left(\frac{C_i}{C_f}\right) = k \times t \quad \text{(S9)}$$

Where $[PMS]_0$ and $[PMS]_f$ are the concentration (mg/L) of PMS at the reaction time of 0 and t (min), respectively. t is reaction time (min), C_i is the initial concentration of MEA, and C_f is the concentration of

MEA after a reaction time of t minutes with the unit of mg/L, respectively.

Text S16. Statistical analysis.

Pre-processing of data: The raw data from catalytic experiments (e.g., concentration measurements from IC spectroscopy) were directly used without mathematical transformation or normalization. In the case of a replicate yielding an obviously aberrant result (e.g., due to operational error) that was inconsistent with the other two replicates, the dataset for that specific condition was re-measured in its entirety to ensure reliability. Data presentation: All quantitative performance data, such as degradation kinetics and removal efficiencies, are presented as the mean ± standard deviation (SD). The error bars in the figures represent this SD. Sample size: All experiments were performed with a minimum of three independent replicates (n = 3) under identical conditions. Statistical methods: The primary statistical approach was descriptive, using the mean and SD to demonstrate data reproducibility and precision. Formal statistical tests for significant differences (e.g., t-tests) were not the focus of this materials-oriented study. Instead, the consistency and small magnitude of the error bars across replicates, combined with the clear performance trends observed (e.g., a marked increase in degradation rate), were used to substantiate the reported enhancements and draw conclusions. Software used for statistical analysis: The calculation of mean values and standard deviations were performed using OriginPro 2023 software.

Figures

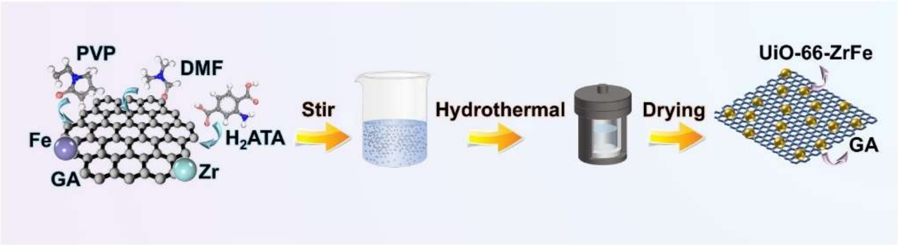

Figure S1. Schematic illustration of the synthetic procedure of UiO-66-ZrFe/GA.

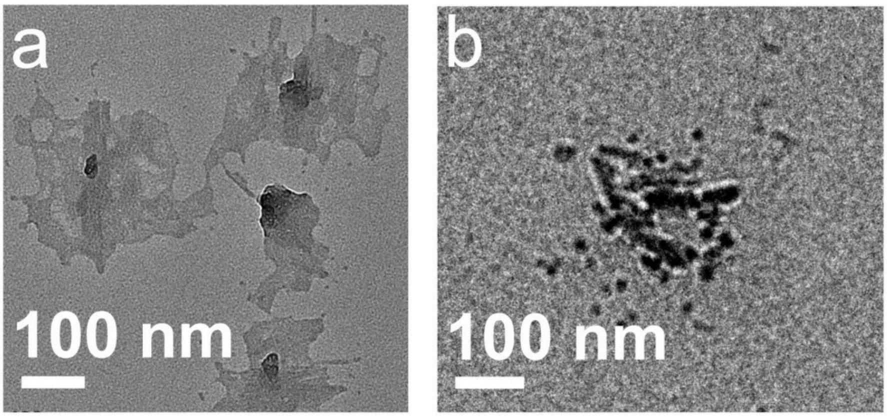

Figure S2. TEM images of (a) GA and (b) UiO-66-ZrFe.

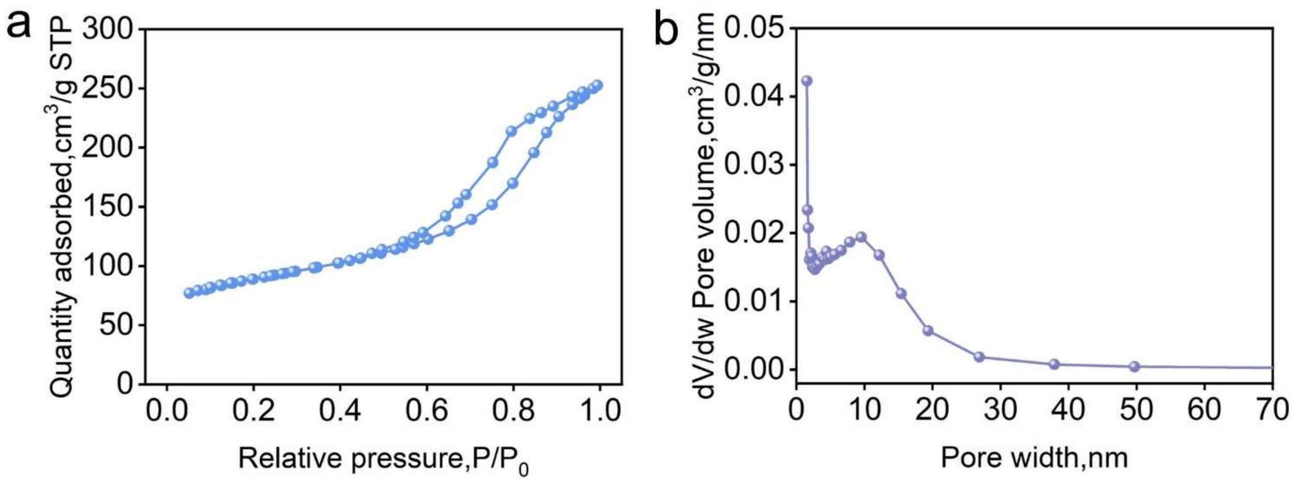

Figure S3. (a) N_2 adsorption/desorption isotherms and (b) pore size distribution of UiO-66-ZrFe/GA.

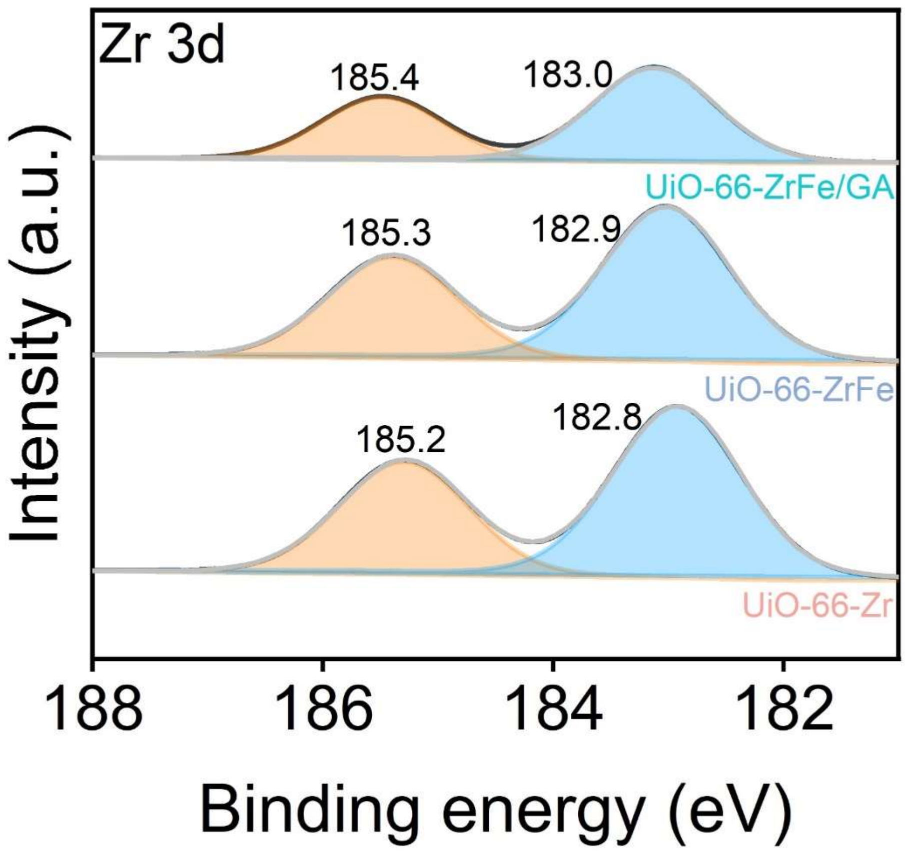

Figure S4. Reproducibility of Zr 3d XPS spectra.

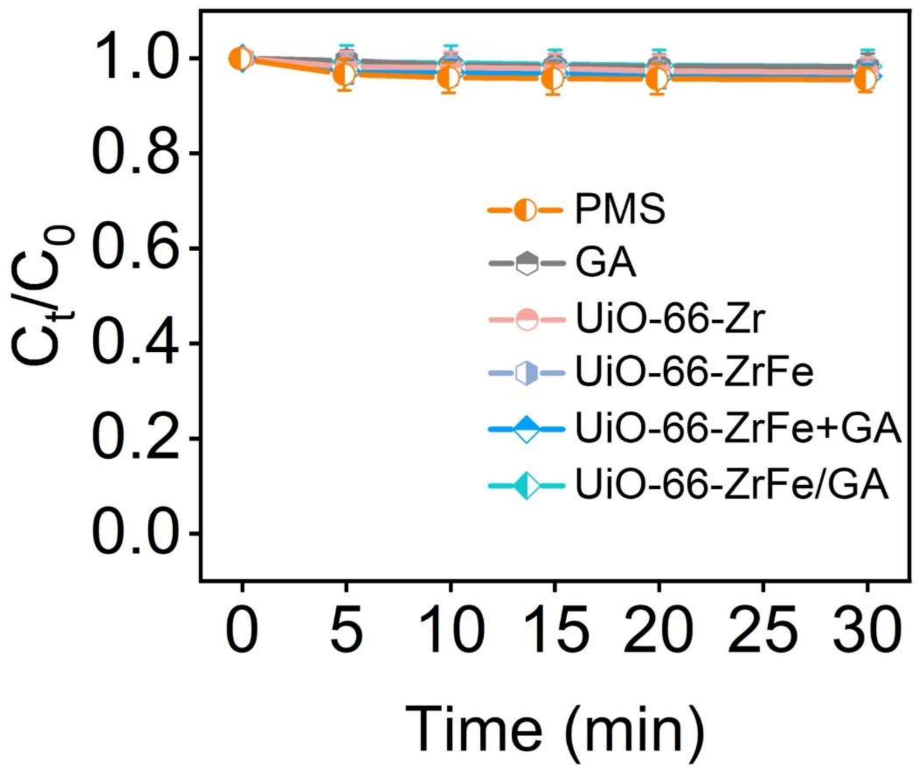

Figure S5. The removal of MEA by catalysts or PMS alone.

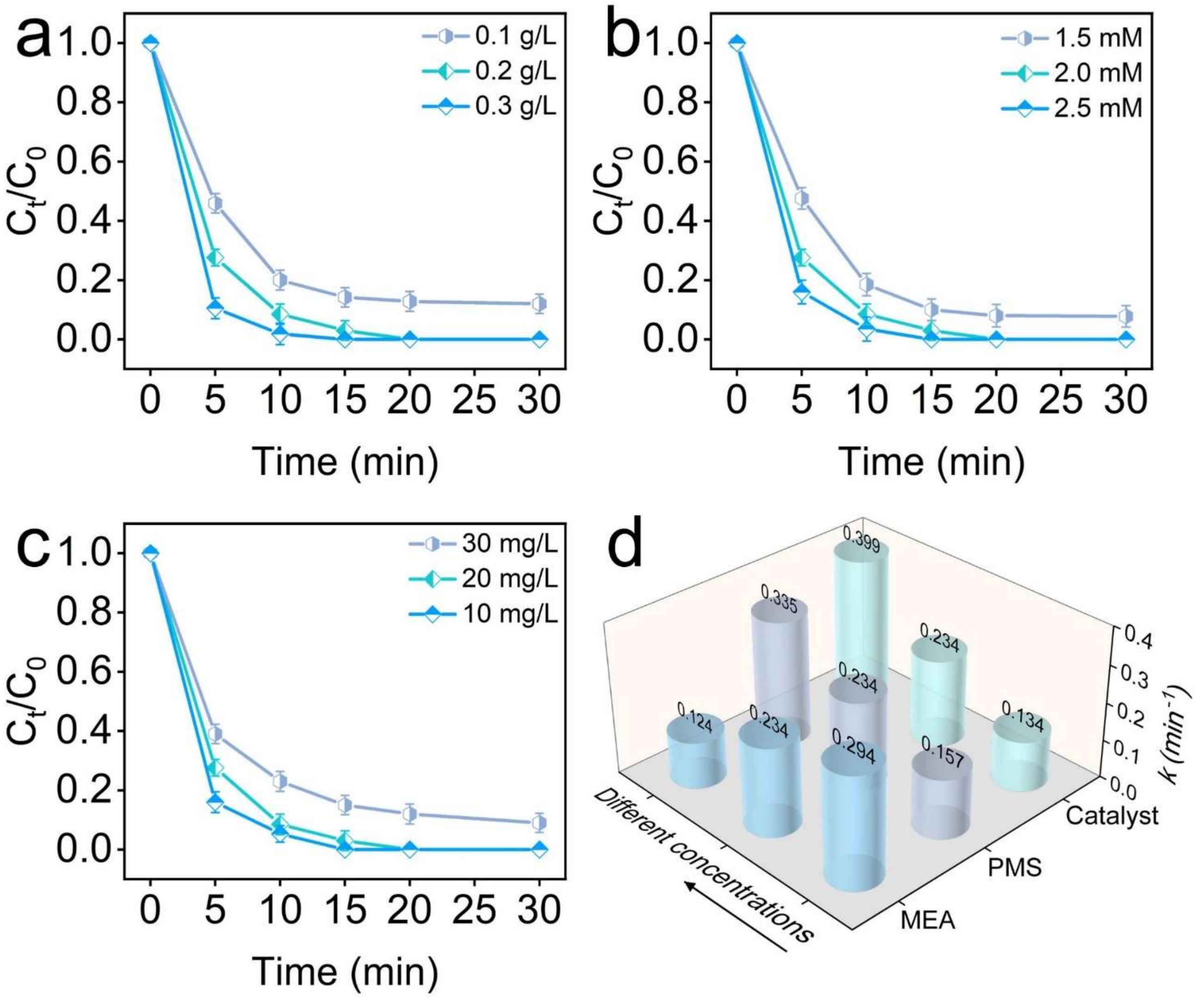

Figure S6. Effects of (a) catalyst dosage, (b) PMS concentrations, and (c) MEA concentrations; (d) the k under various conditions.

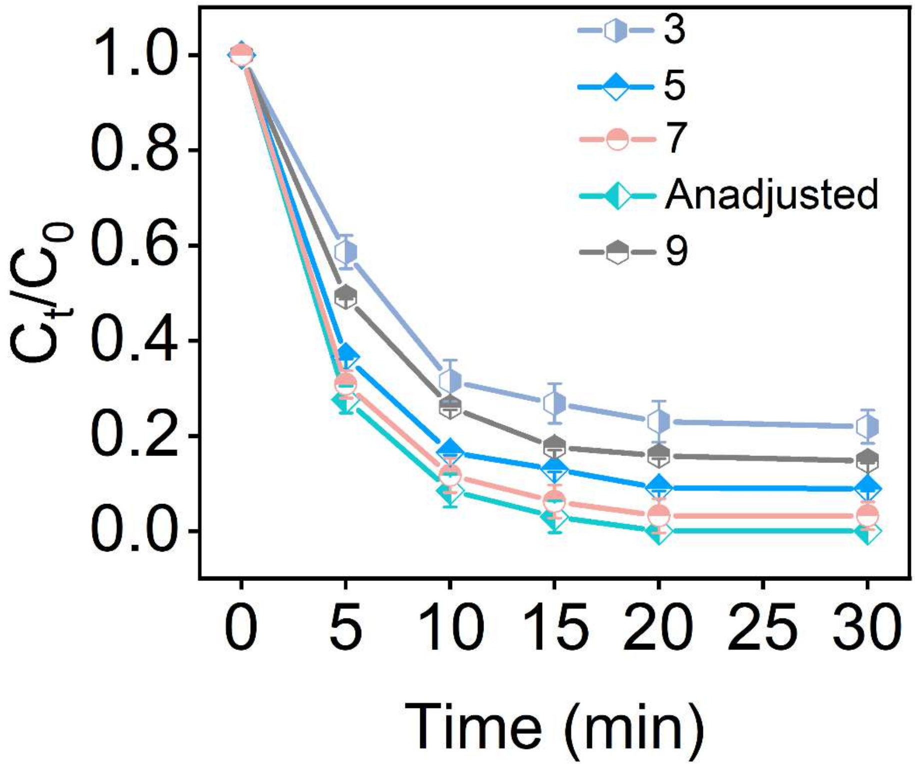

Figure S7. Effects of initial pH.

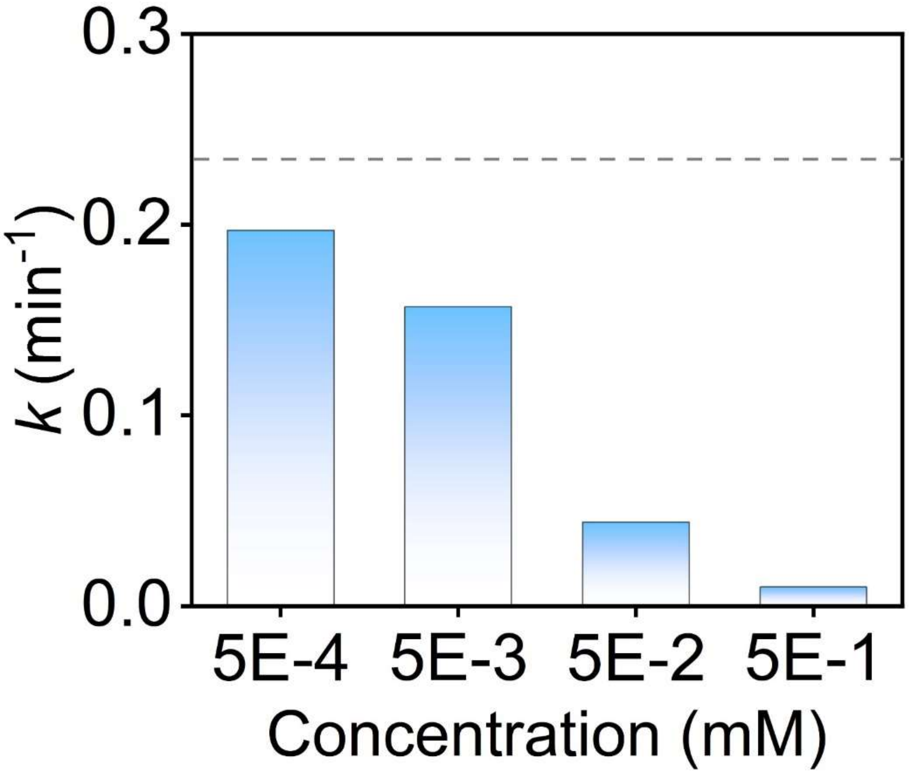

Figure S8. The essential role of Fe(II) validated by ferrozine tests.

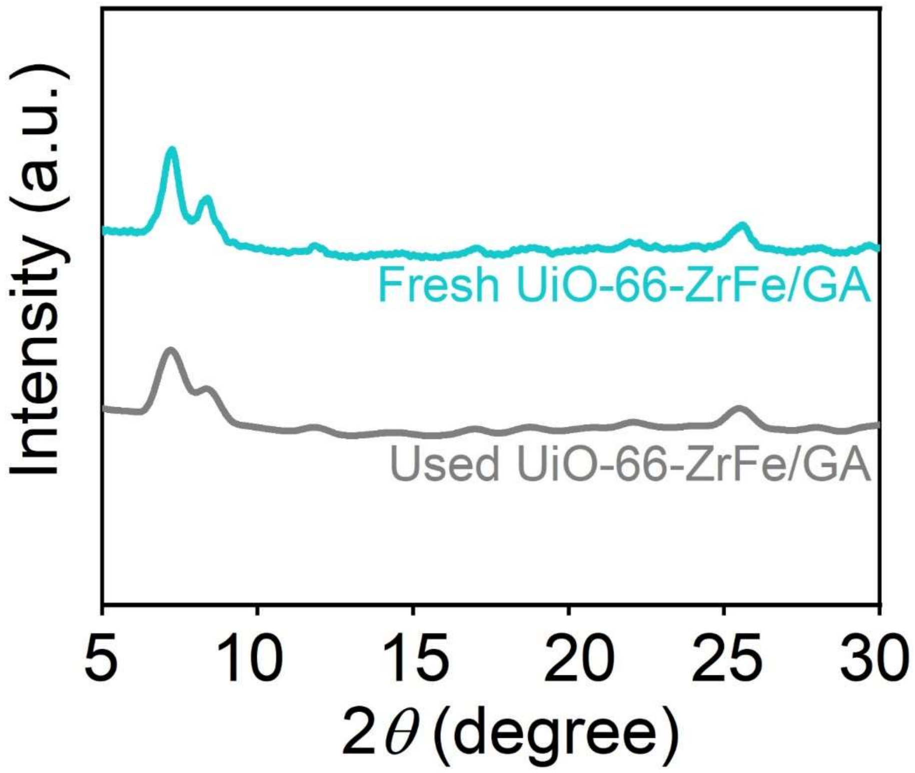

Figure S9. The XRD of fresh and used UiO-66-ZrFe/GA.

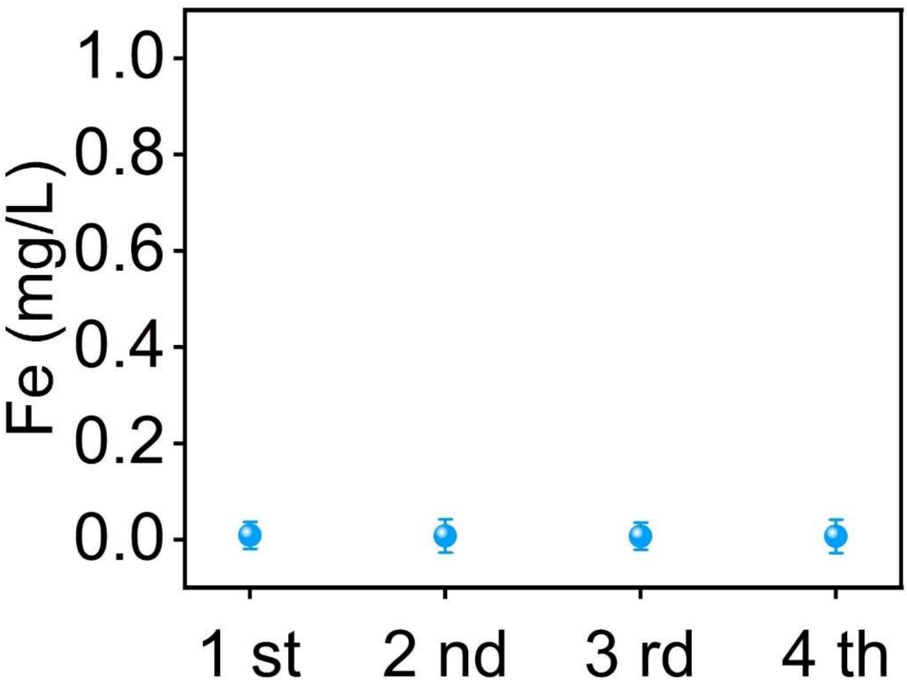

Figure S10. The concentrations of leached Fe during the consecutive cycles.

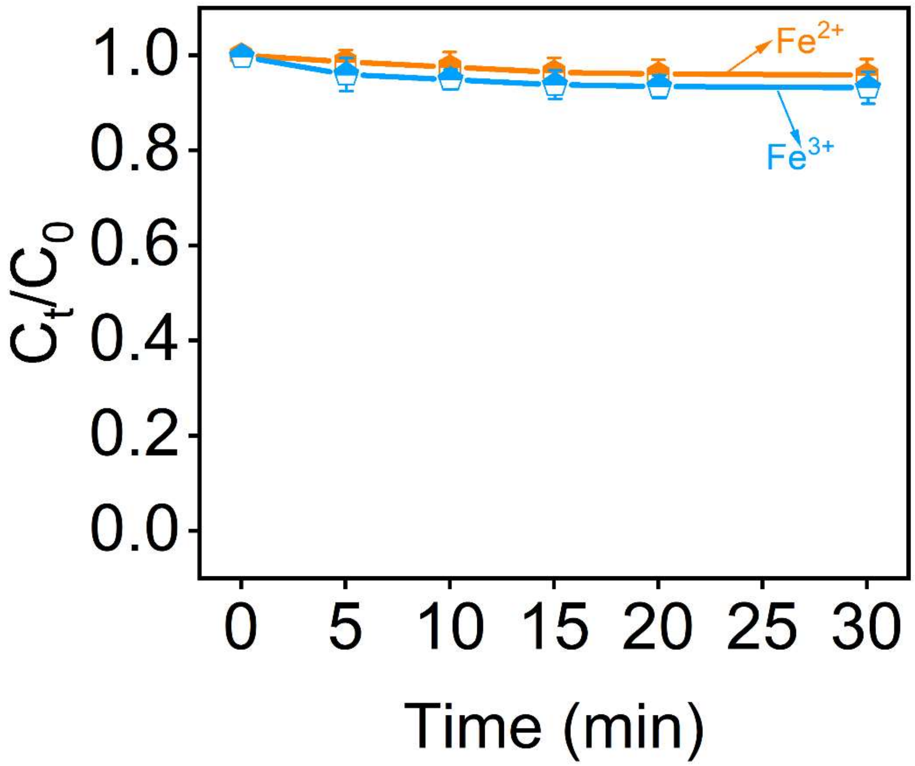

Figure S11. The removal efficiency of MEA in homogeneous systems. Reaction conditions: [Fe^{2+}] = [Fe^{3+}] = 0.01 mg/L.

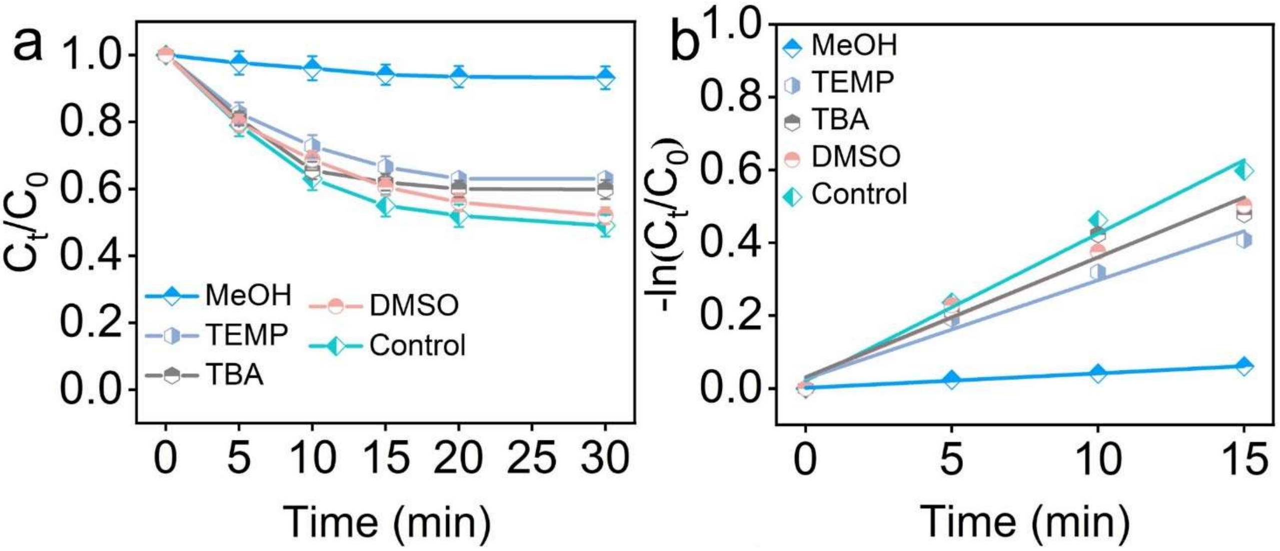

Figure S12. (a) The influences of various scavengers in UiO-66-ZrFe+PMS system and (b) the influences of various scavengers on *k*.

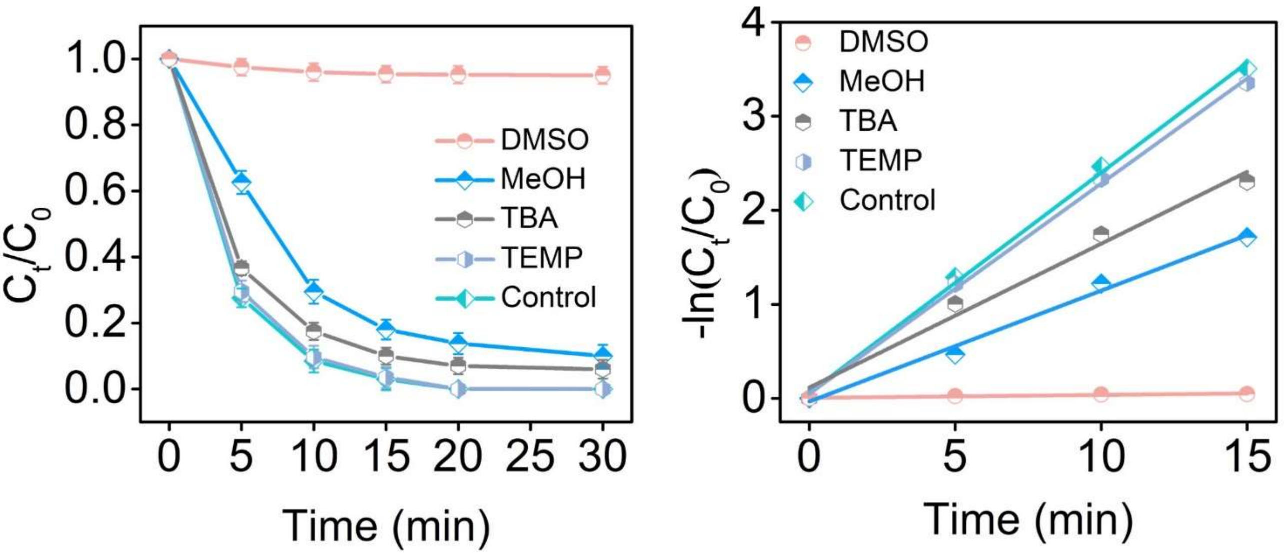

Figure S13. (a) The influences of various scavengers in UiO-66-ZrFe/GA+PMS system and (b) the influences of various scavengers on *k*.

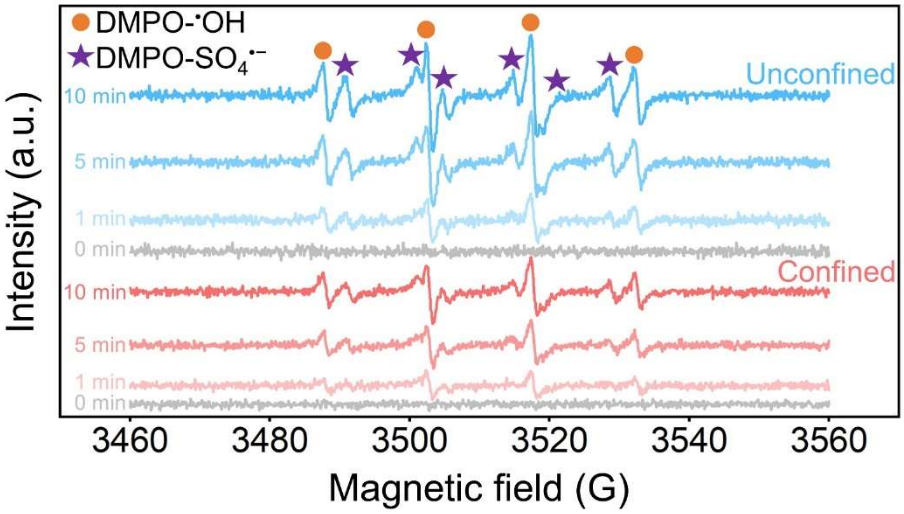

Figure S14. EPR spectra of $SO_4^{\bullet-}$ and $^{\bullet}OH$ in unconfined UiO-66-ZrFe+PMS system and confined UiO-66-ZrFe/GA+PMS system.

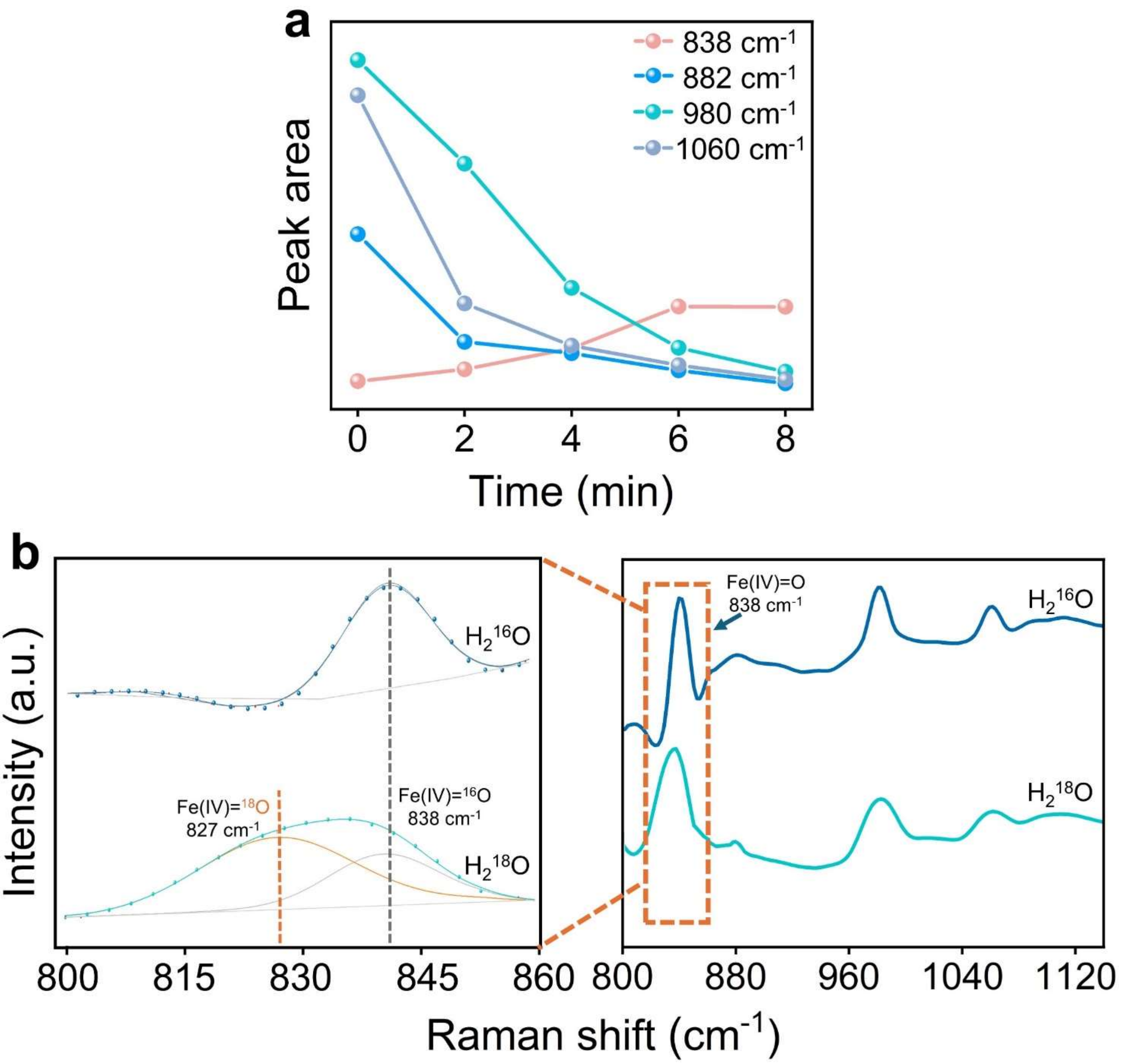

Figure S15. (a) The analysis of integrated peak areas showing the decay of PMS bands and the formation of a steady-state Fe(IV)=O concentration; (b) isotopic shift of the Fe(IV)=O vibration from 838 cm^{-1} in $H_2{}^{16}O$ to 827 cm^{-1} in $H_2{}^{18}O$, providing definitive evidence for the Fe=O bond assignment. These results collectively demonstrate the dynamic steady-state behavior of the Fe(IV)=O intermediate within the confined catalytic microenvironment.

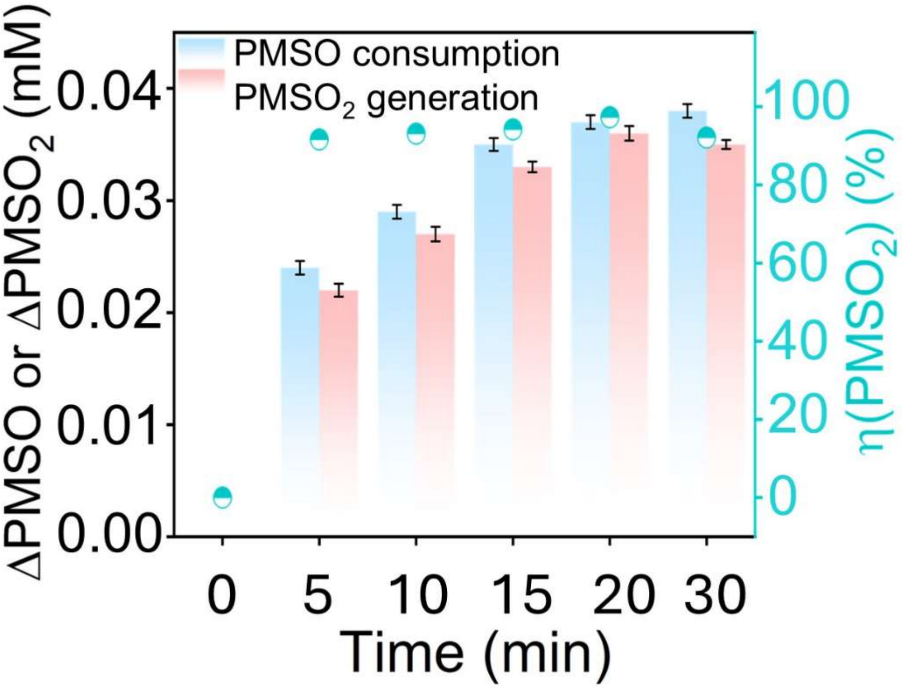

Figure S16. PMSO consumption and $PMSO_2$ generation in UiO-66-ZrFe/GA+PMS system.

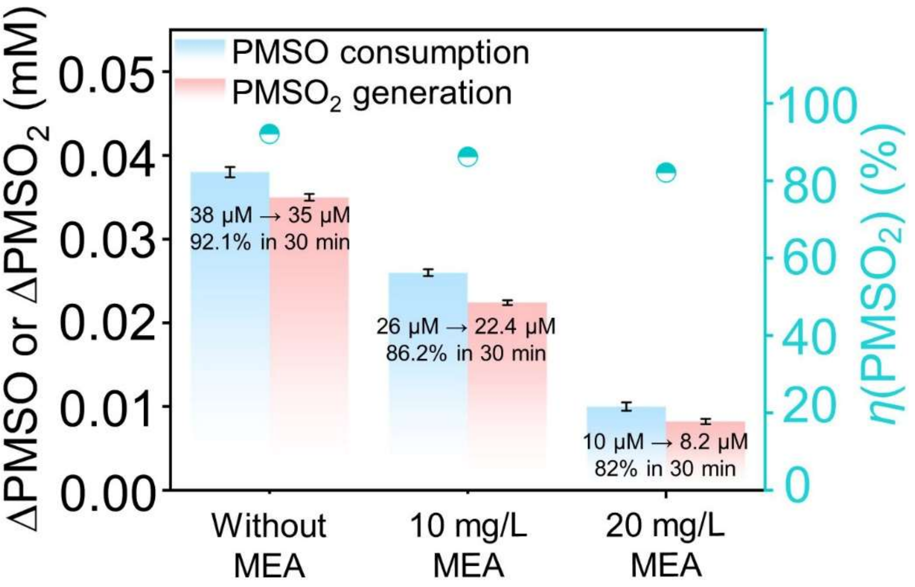

Figure S17. Competition experiment demonstrating the dominance of the Fe(IV)=O pathway.

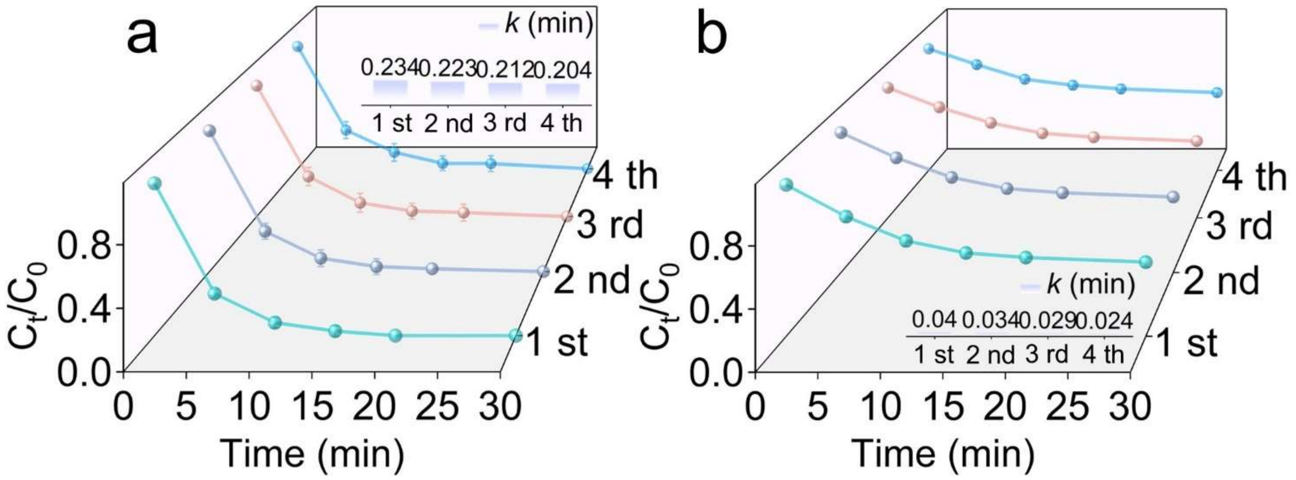

Figure S18. Degradation efficiency and *k* of (a) UiO-66-ZrFe/GA and (b) UiO-66-ZrFe in consecutive cycles.

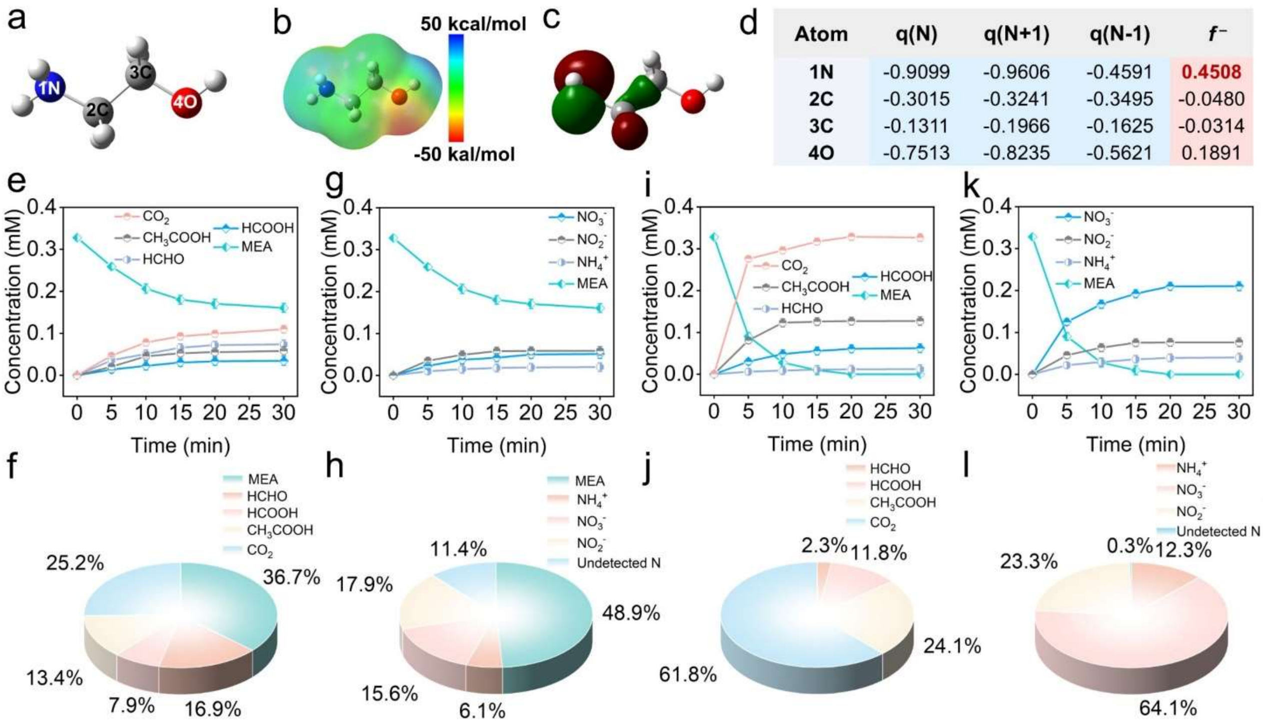

Figure S19. (a) Chemical structure of MEA; (b) ESP; (c) HOMO; (d) Fukui index; (e, f) carbon-containing products and (g, h) nitrogen-containing products in UiO-66-ZrFe+PMS system; (i, j) carbon-containing products and (k, l) nitrogen-containing products in UiO-66-ZrFe/GA+PMS system.

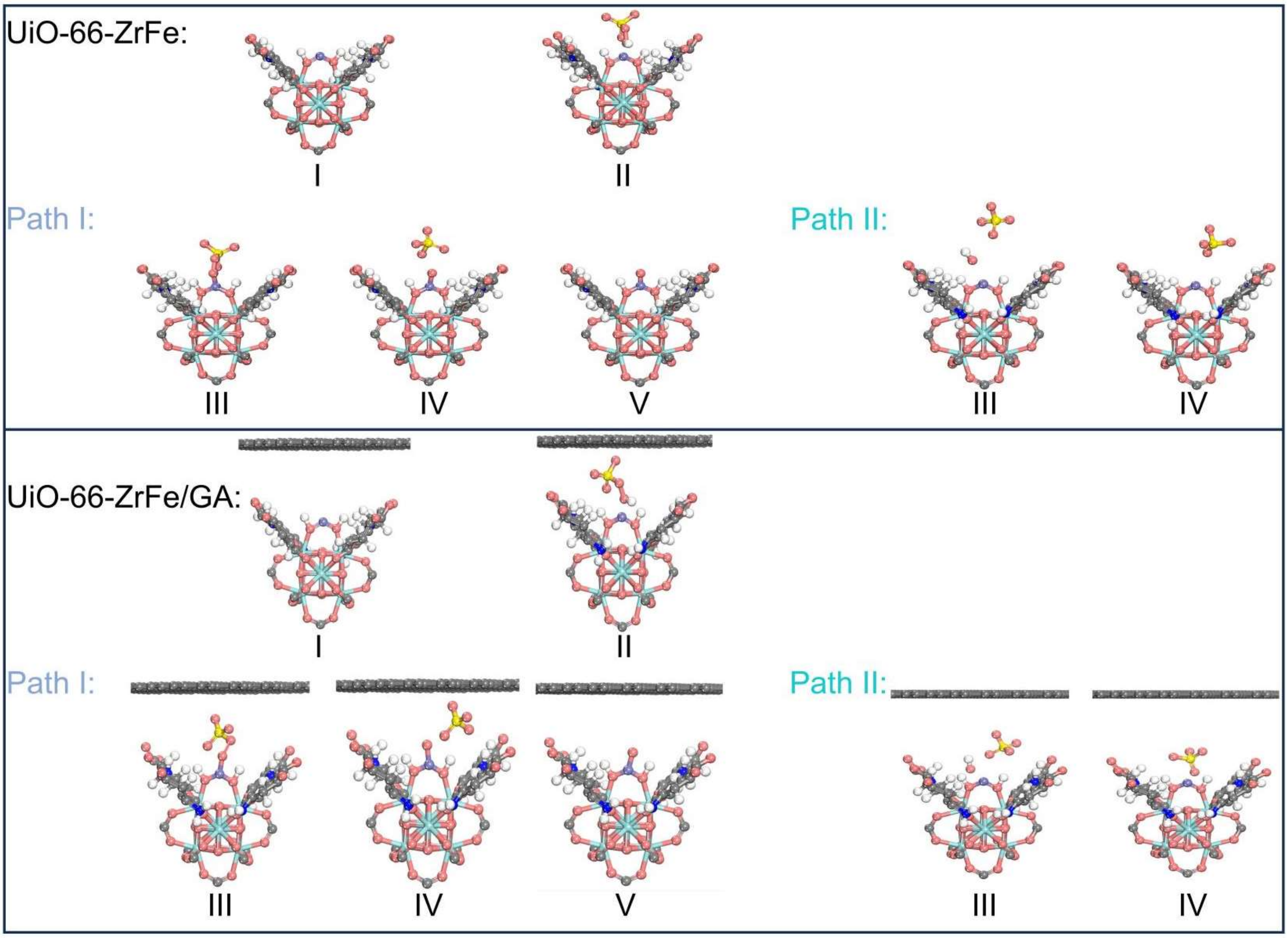

Figure S20. Optimized geometries of reaction intermediates for PMS activation on UiO-66-ZrFe and UiO-66-ZrFe/GA.

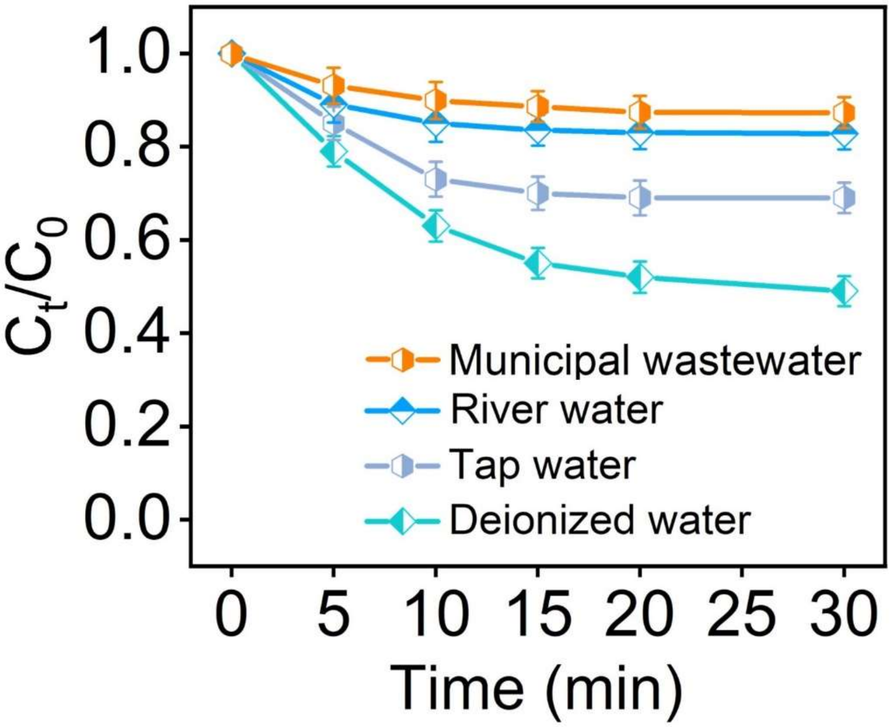

Figure S21. Effects of actual water samples in UiO-66-ZrFe+PMS system.

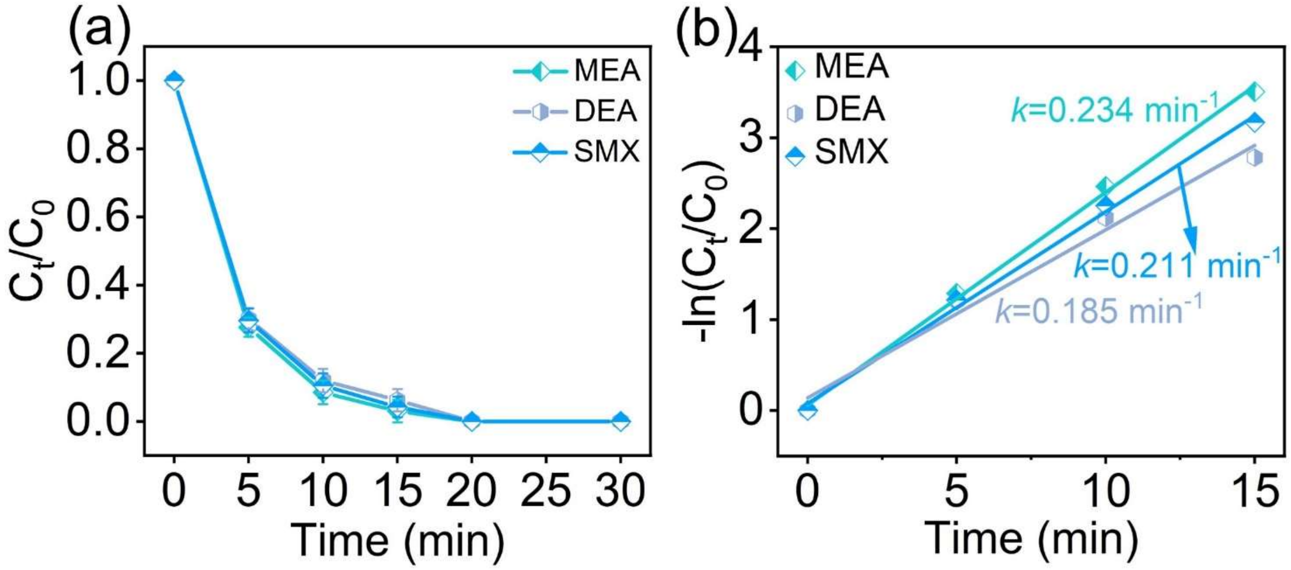

Figure S22. Evaluation of catalytic generality across different pollutant classes. Reaction conditions: $[Pollutant]_0$ = 20 mg/L, [PMS] = 2 mM, catalyst loading = 0.2 g/L, unadjusted pH.

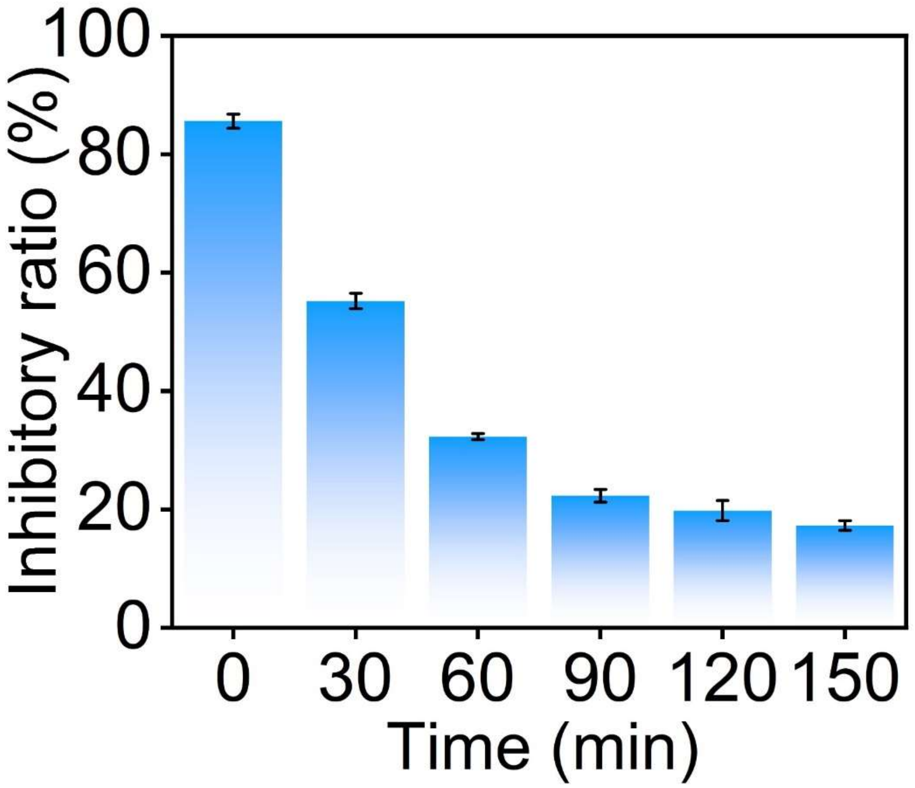

Figure S23. Acute toxicity assessment of the treated solution using a luminescent bacteria (Vibrio fischeri) bioassay.

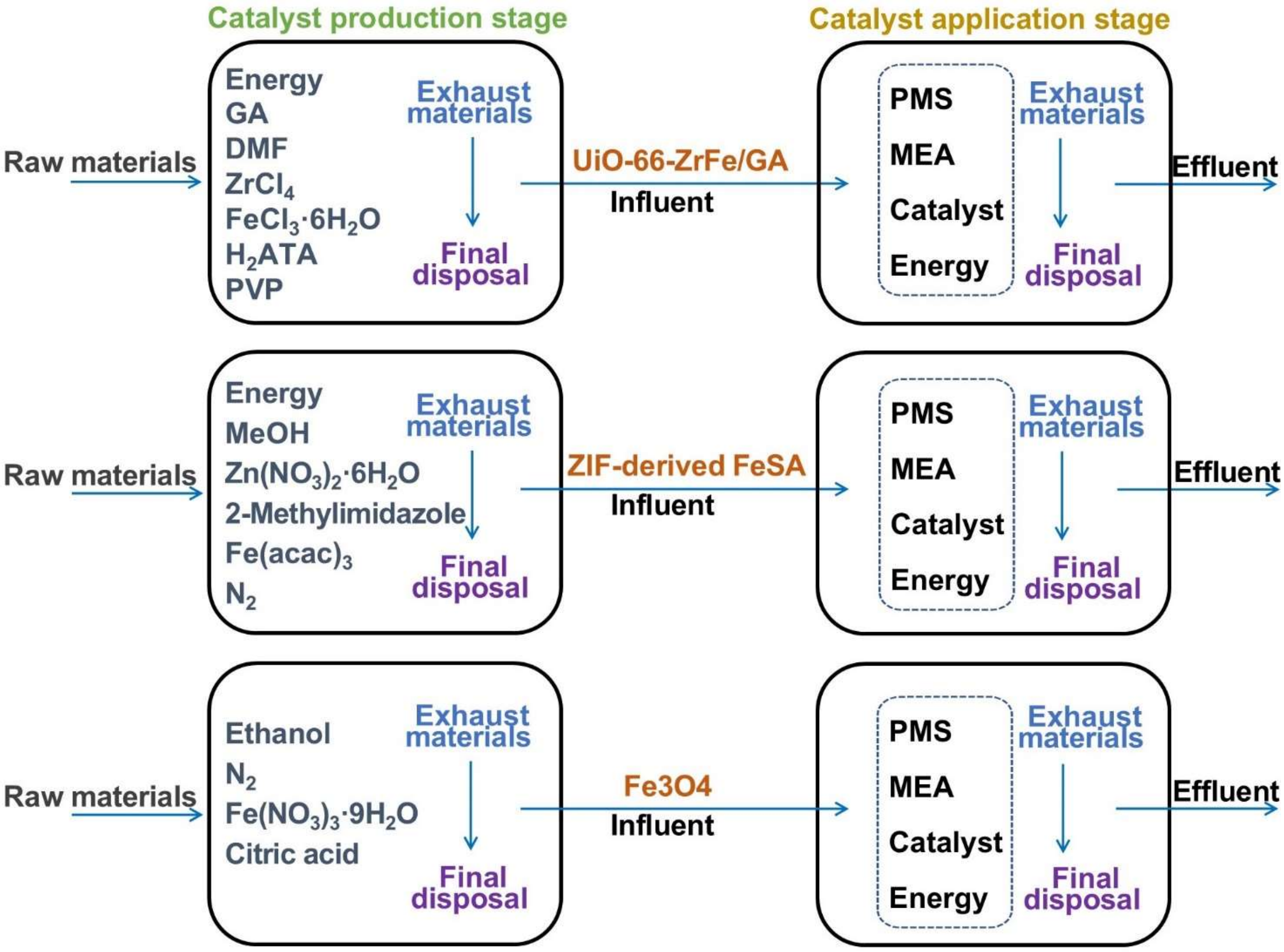

Figure S24. System boundaries for the processes studied in this work.

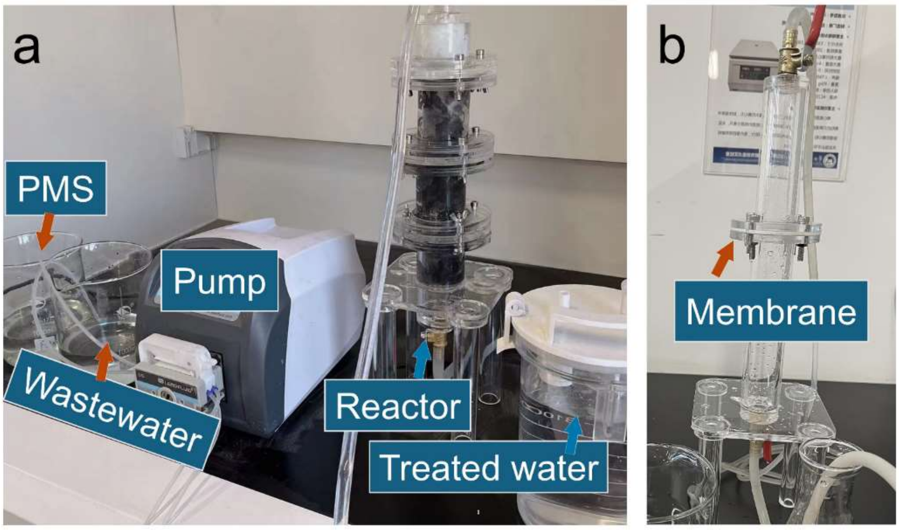

Figure S25. Photographs of the operation of the continuous-flow experiment conducted with (a) an enlarged microreactor and (b) a membrane reactor.

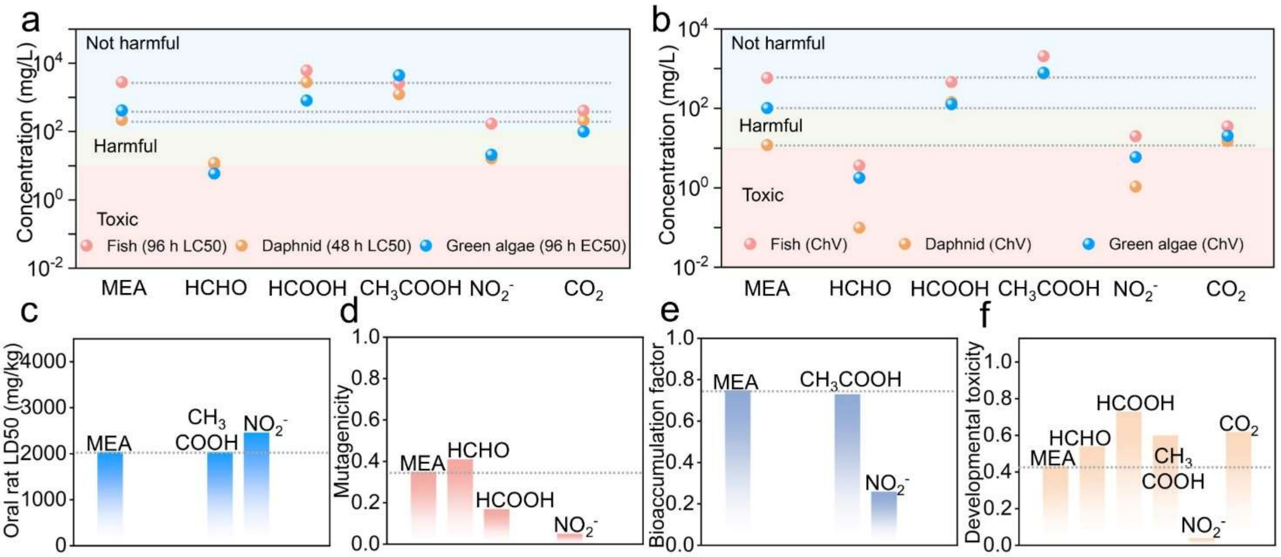

Figure S26. (a) The acute and (b) chronic toxicity obtained from ECOSAR program; (c)–(f) toxicity assessment based on TEST program.

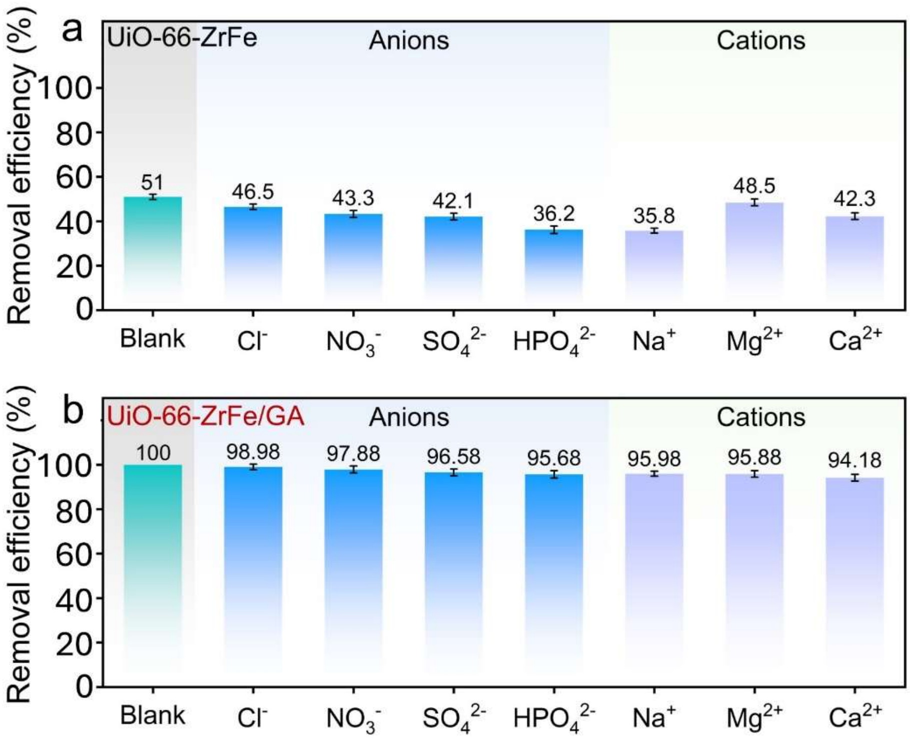

Figure S27. Effects of ions in UiO-66-ZrFe+PMS and UiO-66-ZrFe/GA+PMS systems. The reaction conditions: $[MEA]_0$ = 20 mg/L, [PMS] = 2 mM, [catalyst] = 0.2 g/L, $[ion]_0$ = 3 mM, natural pH.

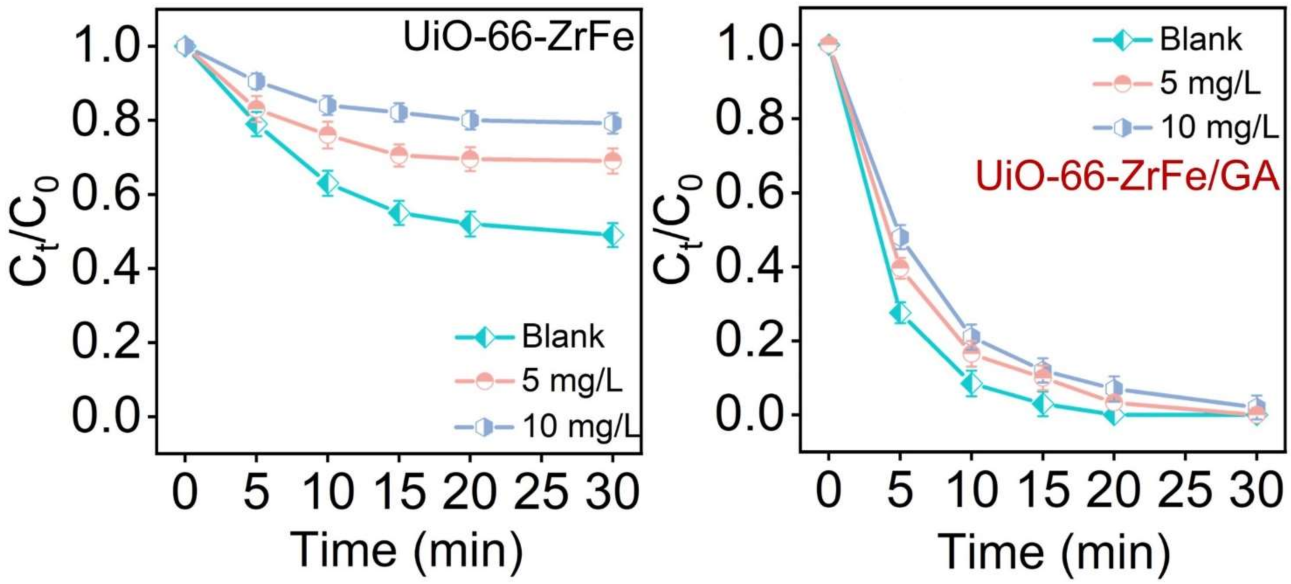

Figure S28. Effects of humic acid in UiO-66-ZrFe+PMS and UiO-66-ZrFe/GA+PMS systems, $[HA]_0$: 5 mg/L and 10 mg/L.

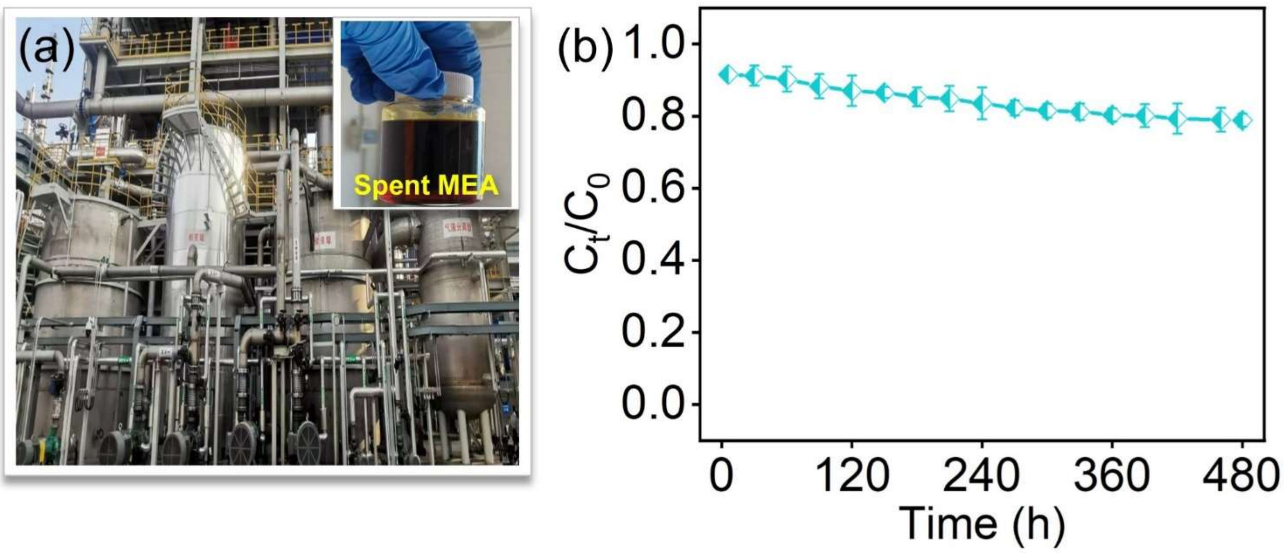

Figure S29. Evaluation of catalytic performance using real industrial waste streams. (a) Photograph of the actual carbon capture plant and the collected spent MEA wastewater used in this study; (b) long-term stability test: MEA removal efficiency by the UiO-66-ZrFe/GA+PMS system over 20 days of continuous operation with the real spent MEA wastewater. Reaction conditions: $[MEA]_0$ = 2 g/L, [PMS] = 200 mM, catalyst loading = 20 g/L.

Tables

Table S1. The HPLC methods and conditions for persistent organic contaminants.

Compounds	Mobile phase	Wavelength (nm)	Flow rate (mL/min)	Temperature (°C)
PMSO	Acetonitrile/0.1% acetic acid=20/80	230	1	35
$PMSO_2$	Acetonitrile/0.1% acetic acid=20/80	215	1	35

Table S2. BET-specific surface area, pore volume, and pore size of UiO-66-ZrFe/GA.

BET surface area (m^2/g)	Pore volume (cm^3/g)	Pore size (nm)
175.18	0.32	6.54

Table S3. EXAFS fitting parameters at the Fe K-edge for various samples (S_0^2=0.84).

Sample	Shell	CN^a	R(Å)^b	σ^2 (Å^2)^c	ΔE_0(eV)^d	R factor
Fe foil	Fe-Fe1	8	2.46±0.01	0.005±0.001	5.41±2.71	0.008
	Fe-Fe2	6	2.85±0.02	0.005±0.001		
FeO	Fe-O	6.02±1.60	2.10±0.06	0.012±0.004	2.55±0.46	0.011
	Fe-Fe	11.75±2.59	3.05±0.08	0.009±0.002		
Fe_2O_3	Fe-O1	2.86±1.07	1.97±0.05	0.003±0.001	2.10±1.02	0.013
	Fe-O2	2.84±1.56	2.15±0.09	0.003±0.00		
	Fe-Fe1	3.19±1.18	3.03±0.03	0.017±0.008		
	Fe-Fe2	3.14±1.18	3.33±0.02	0.008±0.004		
UiO-66-ZrFe	Fe-O	3.84±0.58	1.97±0.01	0.009±0.002	4.42±1.99	0.033
UiO-66-ZrFe/GA	Fe-O	3.94±0.39	1.97±0.01	0.009±0.001	-4.07±1.33	0.006

^{a}*CN*, coordination number; ^{b}*R*, distance between absorber and backscatter atoms; $^c\sigma^2$, Debye-Waller factor to account for both thermal and structural disorders; $^d\Delta E_0$, inner potential correction; *R* factor indicates the goodness of the fit. Fitting range: 3.0 < k (/Å) < 12 and 1.0 < R (Å) < 2.3.

Table S4. The element contents of fresh and used UiO-66-ZrFe/GA catalysts from ICP.

Sample	Zr (wt %)	Fe (wt %)
Fresh UiO-66-ZrFe/GA	2.38%	1.41%
Used UiO-66-ZrFe/GA	2.17%	1.23%

Table S5. Salmonella chromogenic medium in 37℃ train 48 h.

Quality-control strains	Colony color
Pseudomonas aeruginosa	coloriess
Staphylococcus aureus	coloriess
Escherichia coli	green
Salmonella	shiny red
Enterobacter cloacae	blue
Enterobacter aerogenes	blue
Citrobacter freundii	purple

Table S6. Catalytic activity comparison of UiO-66-ZrFe/GA with currently reported Fenton-like catalysts in PMS-mediated pollutant removal.

Catalyst (g L^{-1})	ω_{metal} (wt%)	Δn(pollutants[a]) (mol)	Reaction time (t) (min)	TOF (min^{-1})	Ref.
Fe^{2+} (0.008)	100	TC (20×10^{-6})	15	0.0093	[13]
Fe^{0} (0.05)	100	TC (20×10^{-6})	10	0.0022	[14]
FeO (0.3)	77.7	BPA (10×10^{-6})	30	0.0001	[15]
Fe_3O_4 (0.06)	72.6	APAP (66×10^{-6})	120	0.0007	[16]
Fe_1/ND (0.06)	1.03	TC (30×10^{-6})	40	0.0680	[17]
Fe-SAC (0.1)	0.71	BPA (100×10^{-6})	30	0.2629	[18]
Fe_1/CN (0.2)	6.98	4-CP (78×10^{-6})	10	0.0313	[19]
FeN_x-C-600 (0.2)	5.91	BPA (87×10^{-6})	15	0.0275	[20]
Fe–N_4 (0.06)	0.94	SIZ (20×10^{-6})	15	0.1324	[6b]
Fe_1S/CN (0.5)	9	4-CP (100×10^{-6})	5	0.0249	[21]
Co_{SA}-BNC (0.1)	0.8	SMX (45×10^{-6})	15	0.2213	[22]
Co–N_2 (0.2)	2.26	BPA (50×10^{-6})	5	0.1305	[23]
CoSA (0.15)	2.8	SIZ (20×10^{-6})	8	0.0351	[24]
CoN_1O_2/Mn_3O_4 (0.2)	0.8	SMX (8×10^{-6})	30	0.0098	[25]
SACu@NBC (0.1)	3.41	BPA (87×10^{-6})	30	0.0544	[26]
Cu-In_2O_3/O_v (0.5)	0.98	TC (20×10^{-6})	20	0.0131	[27]
Cu-SA/B-MXene-4 (0.5)	0.86	TC (20×10^{-6})	20	0.0149	[28]
MnN_5 (0.5)	2.8	4-CP (78×10^{-6})	6	0.0511	[29]
Mn-SAC (0.1)	4.07	SMX (40×10^{-6})	20	0.0270	[30]
UiO-66-ZrFe/GA (0.2)	1.41	MEA (16×10^{-6})	20	0.3260	*This work*

[a]Tetracycline hydrochloride (TC); bisphenol A (BPA); acetaminophen (APAP); 4-chlorophenol (4-CP); sulfamethoxazole (SMX); sulfafurazole (SIZ).

Table S7. Performance of the UiO-66-ZrFe/GA+PMS system applied in the enlarged microreactor device for the continuous treatment of actual municipal wastewater samples (water samples were filtered through 0.45 μm membranes before treatment, and the recorded after-treatment data were the parameters after repeated operations).

Parameter	Before treatment	After treatment	Removal efficiency
COD (mg/L)	174.6	20.3-32.5	81.4%-88.4%
TOC (mg/L)	32.5	19.1-19.9	38.8%-41.2%
NH_3-N (mg/L)	65.3	10.3	84.2%
pH	7.9	7.1	

Table S8. Estimation of acute and chronic toxicity of MEA and its transformation products to fish, daphnid, and green algae using the ECOSAR program.

Compound	Acute toxicity (mg/L)			Chronic toxicity (ChV) (mg/L)		
	Fish (LC_{50})	Daphnid (LC_{50})	Green algae (EC_{50})	Fish	Daphnid	Green algae
MEA	19400	1790	2440	2460	114	675
HCHO	965	85.1	127	141	5.22	34
HCOOH	25600	2310	3310	3500	144	899
CH_3COOH	1800000	118000	317000	645000	5420	68400
NO_2^-	45.8	5.58	4.40	2.49	0.468	1.49
CO_2	146000	11500	21600	30300	631	5320

Notes: LC_{50}: Median lethal concentration. A statistically derived concentration of a substance that can be expected to cause death in 50% of test animals. It is usually expressed as milligrams (mg) of substance per liter (L) water.

EC_{50}: Median effect concentration. A statistically derived concentration of a substance that can be expected to cause a specific effect (e.g., growth inhibition) in 50% of test animals. It is usually expressed as milligrams (mg) of substance per liter (L) water.

ChV, Chronic value, represents chronic toxicity. ChV is defined as the geometric mean of the no observed effect concentration and the lowest observed effect concentration.

Table S9. Estimation of the Oral rat LD50, mutagenicity, bioaccumulation factor, and developmental toxicity for MEA and its transformation products using the TEST program.

Compound	Oral rat LD50 (mg/kg)	Mutagenicity		Bioaccumulation factor	Developmental toxicity	
		value	result		value	result
MEA	2037.22	0.35	negative	0.75	0.43	non-toxicant
HCHO	--	0.41	negative	--	0.54	toxicant
HCOOH	--	0.17	negative	--	0.73	toxicant
CH_3COOH	2033.69	-0.02	negative	0.73	0.60	toxicant
NO_2^-	2461.07	0.05	negative	0.26	0.04	non-toxicant
CO_2	--	--	--	--	0.62	toxicant

Table S10. Input materials and energy required in the catalyst production stage to degrade 1 kg MEA through Fe_3O_4/PMS, ZIF-derived FeSA/PMS and UiO-66-ZrFe/GA+PMS processes.

Catalyst production stage	Input	Amount	Unit	Output	Amount	Unit
Stage 1 (UiO-66-ZrFe/GA)	Heat for preparation	320	kwh	Intermediates (Catalyst precursors)	3	kg
	GO	3	kg	Exhausted volatile substance	24	kg
	EDA	3	kg	Exhausted MeOH	800	L
	MeOH	800	L			
Stage 2 (UiO-66-ZrFe/GA)	GA	3	kg	Intermediates (Catalyst)	10	kg
	$FeCl_3 \cdot 6H_2O$	27	kg	Exhausted volatile substance	6	kg
	$ZrCl_4$	23.3	kg			
	H_2ATA	36.2	kg			
	Heat for preparation	450	kwh			
	PVP	20	kg			
	DMF	4000	kg			
	MeOH	800	L			
Stage 1 (ZIF-derived FeSA)	Heat for preparation	1719	kwh	Intermediates (Catalyst precursors)	108	kg
	$Zn(NO_3)_2 \cdot 6H_2O$	690	kg	Exhausted materials	2142	kg
	2-Methylimidazole	1500	kg	Exhausted MeOH	3450	L
	$Fe(NO_3)_3 \cdot 9H_2O$	60	kg			
	MeOH	3450	L			
Stage 2 (ZIF-derived FeSA)	Catalyst precursors	108	kg	Intermediates (Catalyst)	42	kg
	Heat for preparation	1560	kwh	Exhausted volatile substance	72	kg
	N_2	10350	L			
Stage 1 (Fe_3O_4)	Heat for preparation	975	kwh	Intermediates (Catalyst precursors)	360	kg
	Citric acid	90	kg	Exhausted materials	150	kg
	$Fe(NO_3)_3 \cdot 9H_2O$	420	kg	Exhausted ethanol	270	L

	Ethanol	270	L			
Stage 2 (Fe_3O_4)	Catalyst precursors	360	kg	Intermediates (Catalyst)	240	kg
	Heat for preparation	7800	kwh	Exhausted volatile substance	120	kg
	N_2	43200	L			

Calculations based on catalytic activity showed that 10 kg of UiO-66-ZrFe/GA or 36 kg of FeSA or 240 kg of Fe_3O_4 were required to treat 1 kg MEA, respectively.

Table S11. Comparative cost analysis of three catalysts.

Catalyst 1: UiO-66-ZrFe/GA				
Item / Energy	Quantity	Unit price (USD)	Subtotal (USD)	Notes
**Raw Materials**				
GO	3 kg	41.67	125	High purity
EDA	3 kg	3.47	10.41	Industrial grade
MeOH	1600 L	0.49	784	Recyclable solvent
$FeCl_3 \cdot 6H_2O$	27 kg	0.56	15.12	Industrial grade
$ZrCl_4$	23.3 kg	25	582.5	**Major cost driver**
H_2ATA	36.2 kg	2.08	75.3	
PVP	20 kg	11.11	222.2	
DMF	4000 kg	1.67	6680	Recyclable solvent
**Energy**				
Heating	770 kWh	0.11	84.7	Industrial electricity
**Total**			**8579.23**	

Catalyst 2: ZIF-derived FeSA				
Item / Energy	Quantity	Unit Price (USD)	Subtotal (USD)	Notes
**Raw Materials**				
$Zn(NO_3)_2 \cdot 6H_2O$	690 kg	1.39	959.1	
2-Methylimidazole	1500 kg	16.67	25005	**Major cost driver**

$Fe(NO_3)_3 \cdot 9H_2O$	60 kg	0.97	58.2	
MeOH	3450 L	0.49	1690.5	Recyclable solvent
Catalyst precursors	108 kg	5.56	600.48	Estimated
**Energy**				
Heating	3279 kWh	0.11	360.69	
N_2 gas	10350 L	0.03	310.5	
**Total**			**28984.47**	

Catalyst 3: Fe_3O_4

Item / Energy	Quantity	Unit Price (USD)	Subtotal (USD)	Notes
**Raw Materials**				
Citric acid	90 kg	1.11	99.99	
$Fe(NO_3)_3 \cdot 9H_2O$	420 kg	0.97	407.4	
Ethanol	270 L	0.83	224.1	Recyclable solvent
Catalyst precursors	360 kg	2.78	1000.8	Estimated
**Energy**				
Heating	8775 kWh	0.11	965.25	**High energy demand**
N_2 gas	43200 L	0.03	1296	**Significant gas use**
**Total**			**3993.54**	

References

[1] X. Zhang, J. Tang, L. Wang, C. Wang, L. Chen, X. Chen, J. Qian, B. Pan, Nanoconfinement-triggered oligomerization pathway for efficient removal of phenolic pollutants via a Fenton-like reaction, *Nat. Commun.* **2024**, 15, 917.

[2] L. Zhang, J. Qi, W. Chen, X. Yang, Z. Fang, J. Li, X. Li, S. Lu, L. Wang, Constructing hollow multishelled microreactors with a nanoconfined microenvironment for ofloxacin degradation through peroxymonosulfate activation: Evolution of high-valence cobalt-oxo species, *Environ. Sci. Technol.* **2023**, 57, 16141.

[3] Z. Fang, J. Qi, W. Chen, L. Zhang, J. Wang, C. Tian, Q. Dai, W. Liu, L. Wang, Defect engineering-mediated Co_9S_8 with unexpected catalytic selectivity for heterogeneous Fenton-like reaction: Unveiling the generation route of $^1O_2$ in V_S active site, *Appl. Catal., B* **2023**, 338, 123084.

[4] Z. Fang, J. Qi, Y. Xu, Y. Liu, T. Qi, L. Xing, Q. Dai, L. Wang, Promoted generation of singlet oxygen by hollow-shell CoS/g-C_3N_4 catalyst for sulfonamides degradation, *Chem. Eng. J.* **2022**, 441, 136051.

[5] L. Zhang, J. Qi, H. Zhang, L. Xing, Z. Zhou, R. Lei, B. Li, H. Liu, L. Wang, Dual sites design of CoFe/Mg MMO catalyst for effectively suppressing dimethylamine re-emission: Degradation products and DFT calculation, *Chem. Eng. J.* **2023**, 457, 141223.

[6] a) Q. Yang, X. Yang, Y. Yan, C. Sun, H. Wu, J. He, D. Wang, Heterogeneous activation of peroxymonosulfate by different ferromanganese oxides for tetracycline degradation: Structure dependence and catalytic mechanism, *Chem. Eng. J.* **2018**, 348, 263; b) Z. Wu, B. Huang, X. Wang, C.-S. He, Y. Liu, Y. Du, W. Liu, Z. Xiong, B. Lai, Facilely tuning the first-shell coordination microenvironment in iron single-atom for Fenton-like chemistry toward highly efficient wastewater purification, *Environ. Sci. Technol.* **2023**, 57, 14046.

[7] a) D. Ma, J. Wang, K. Feng, B. Liu, G. Xie, D. Xing, A green strategy from waste red mud to Fe^0-based biochar for sulfadiazine treatment by peroxydisulfate activation, *Chem. Eng. J.* **2022**, 446, 136944; b) T. Liu, S. Xiao, N. Li, J. Chen, X. Zhou, Y. Qian, C.-H. Huang, Y. Zhang, Water decontamination via nonradical process by nanoconfined Fenton-like catalysts, *Nat. Commun.* **2023**, 14, 2881.

[8] a) H. Yao, P. Sun, D. Minakata, J. C. Crittenden, C.-H. Huang, Kinetics and modeling of degradation of Ionophore antibiotics by UV and UV/H_2O_2, *Environ. Sci. Technol.* **2013**, 47, 4581; b) D. He, Y. Cheng, Y. Zeng, H. Luo, K. Luo, J. Li, X. Pan, D. Barceló, J. C. Crittenden, Synergistic activation of peroxymonosulfate and persulfate by ferrous ion and molybdenum disulfide for pollutant degradation: Theoretical and experimental studies, *Chemosphere* **2020**, 240, 124979.

[9] R. Guo, Y. Zhu, X. Cheng, J. Li, J. C. Crittenden, Efficient degradation of lomefloxacin by Co-Cu-LDH activating peroxymonosulfate process: Optimization, dynamics, degradation pathway and mechanism, *J. Hazard. Mater.* **2020**, 399, 122966.

[10] M. G. Antoniou, I. Boraei, M. Solakidou, Y. Deligiannakis, M. Abhishek, L. A. Lawton, C. Edwards, Enhancing photocatalytic degradation of the cyanotoxin microcystin-LR with the addition of sulfate-radical generating oxidants, *J. Hazard. Mater.* **2018**, 360, 461.

[11] Y. Lee, S. Lee, M. Cui, Y. Ren, B. Park, J. Ma, Z. Han, J. Khim, Activation of peroxodisulfate and peroxymonosulfate by ultrasound with different frequencies: Impact on ibuprofen removal efficient, cost estimation and energy analysis, *Chem. Eng. J.* **2021**, 413, 127487.

[12] S. Jung, S. Lee, X. Dou, E. E. Kwon, Valorization of disposable COVID-19 mask through the thermo-chemical process, *Chem. Eng. J.* **2021**, 405, 126658.

[13] H. Cheng, H. Liu, C. Huang, J. Xu, H. Tian, J. Yang, P. Wang, J. Cai, M. Cheng, Z. Liu, Tungsten carbide induced acceleration of Fe^{3+}/Fe^{2+} cycle in Fe^{2+}/PMS process for rapid degradation of tetracycline hydrochloride, *Sep. Purif. Technol.* **2024**, 330, 125311.

[14] J. Cao, L. Lai, B. Lai, G. Yao, X. Chen, L. Song, Degradation of tetracycline by peroxymonosulfate activated with zero-valent iron: Performance, intermediates, toxicity and mechanism, *Chem. Eng. J.* **2019**, 364, 45.

[15] B. Zeng, L. Long, Y. Chen, Z. Liu, L. Luo, Q. Shao, P. Xie, J. Ma, New insights into the FeO/peroxymonosulfate process for organics removal: The synergistic effect of radicals and Fe(IV), *ACS ES&T Eng.* **2023**, 3, 1886.

[16] C. Tan, N. Gao, Y. Deng, J. Deng, S. Zhou, J. Li, X. Xin, Radical induced degradation of acetaminophen with Fe_3O_4 magnetic nanoparticles as heterogeneous activator of peroxymonosulfate, *J. Hazard. Mater.* **2014**, 276, 452.

[17] M. Yang, K. Wu, S. Sun, J. Duan, X. Liu, J. Cui, S. Liang, Y. Ren, Unprecedented relay catalysis of curved $Fe_1–N_4$ single-atom site for remarkably efficient $^1O_2$ generation, *ACS Catal.* **2023**, 13, 681.

[18] Y. Gao, T. Wu, C. Yang, C. Ma, Z. Zhao, Z. Wu, S. Cao, W. Geng, Y. Wang, Y. Yao, Y. Zhang, C. Cheng, Activity trends and mechanisms in peroxymonosulfate-assisted catalytic production of singlet oxygen over atomic metal-N-C catalysts, *Angew. Chem., Int. Ed.* **2021**, 60, 22513.

[19] L.-S. Zhang, X.-H. Jiang, Z.-A. Zhong, L. Tian, Q. Sun, Y.-T. Cui, X. Lu, J.-P. Zou, S.-L. Luo, Carbon nitride supported high-loading Fe single-atom catalyst for activation of peroxymonosulfate to generate $^1O_2$ with 100 % selectivity, *Angew. Chem., Int. Ed.* **2021**, 60, 21751.

[20] B. Zhang, X. Li, K. Akiyama, P. A. Bingham, S. Kubuki, Elucidating the mechanistic origin of a spin state-dependent FeN_x–C catalyst toward organic contaminant oxidation via peroxymonosulfate activation, *Environ. Sci. Technol.* **2022**, 56, 1321.

[21] X. Jiang, B. Zhou, W. Yang, J. Chen, C. Miao, Z. Guo, H. Li, Y. Hou, X. Xu, L. Zhu, D. Lin, J. Xu, Precise coordination of high-loading Fe single atoms with sulfur boosts selective generation of nonradicals, *Proc. Natl. Acad. Sci.* **2024**, 121, e2309102121.

[22] J. Song, N. Hou, X. Liu, M. Antonietti, P. Zhang, R. Ding, L. Song, Y. Wang, Y. Mu, Asymmetrically coordinated CoB_1N_3 moieties for selective generation of high-valence Co-oxo species via coupled electron–proton transfer in Fenton-like reactions, *Adv. Mater.* **2023**, 35, 2209552.

[23] X. Liang, D. Wang, Z. Zhao, T. Li, Y. Gao, C. Hu, Coordination number dependent catalytic activity of single-atom cobalt catalysts for Fenton-like reaction, *Adv. Funct. Mater.* **2022**, 32, 2203001.

[24] Z. Wu, Z. Xiong, W. Liu, R. Liu, X. Feng, B. Huang, X. Wang, Y. Gao, H. Chen, G. Yao, B. Lai, Active center size-dependent Fenton-like chemistry for sustainable water decontamination, *Environ. Sci. Technol.* **2023**, 57, 21416.

[25] X. Li, X. Wen, J. Lang, Y. Wei, J. Miao, X. Zhang, B. Zhou, M. Long, P. J. J. Alvarez, L. Zhang, CoN_1O_2 single-atom catalyst for efficient peroxymonosulfate activation and selective cobalt(IV)=O generation, *Angew. Chem., Int. Ed.* **2023**, 62, e202303267.

[26] J. Pan, B. Gao, P. Duan, K. Guo, M. Akram, X. Xu, Q. Yue, Y. Gao, Improving peroxymonosulfate activation by copper ion-saturated adsorbent-based single atom catalysts for the degradation of organic contaminants: electron-transfer mechanism and the key role of Cu single atoms, *J. Mater. Chem. A* **2021**, 9, 11604.

[27] Z. Zhao, P. Wang, C. Song, T. Zhang, S. Zhan, Y. Li, Enhanced interfacial electron transfer by asymmetric Cu-O_v-In sites on In_2O_3 for efficient peroxymonosulfate activation, *Angew. Chem., Int. Ed.* **2023**, 62, e202216403.

[28] P. Yang, Z. Cao, Y. Long, D. Liu, W. Huang, S. Zhan, M. Li, Regulating the local electronic structure of copper single atoms with unsaturated B,O-coordination for selective $^1O_2$ generation, *ACS Catal.* **2023**, 13, 12414.

[29] J. Miao, J. Song, J. Lang, Y. Zhu, J. Dai, Y. Wei, M. Long, Z. Shao, B. Zhou, P. J. J. Alvarez, L. Zhang, Single-atom MnN_5 catalytic sites enable efficient peroxymonosulfate activation by forming highly reactive Mn(IV)–oxo species, *Environ. Sci. Technol.* **2023**, 57, 4266.

[30] K. Yin, Y. Shang, D. Chen, B. Gao, Q. Yue, X. Xu, Redox potentials of pollutants determining the dominate oxidation pathways in manganese single-atom catalyst (Mn-SAC)/peroxymonosulfate system: Selective catalytic mechanisms for versatile pollutants, *Appl. Catal., B* **2023**, 338, 123029.
